# Finite Element Analysis for the Self-Loosening Behavior of the Bolted Joint with a Superelastic Shape Memory Alloy

**DOI:** 10.3390/ma11091592

**Published:** 2018-09-02

**Authors:** Xiangjun Jiang, Jin Huang, Yongkun Wang, Baotong Li, Jingli Du, Peng Hao

**Affiliations:** 1 Key Laboratory of Electronic Equipment Structural Design, Xidian University, Xi’an 710071, China; xjjiang@xidian.edu.cn (X.J.); ykwang@xidian.edu.cn (Y.W.); jldu@mail.xidian.edu.cn (J.D.); 2 State Key Laboratory for Manufacturing System, Xi’an Jiaotong University, Xi’an 710049, China; baotong.me@mail.xjtu.edu.cn; 3 State Key Laboratory of Structural Analysis for Industrial Equipment, Dalian University of Technology, Dalian 116023, China; haopeng@dlut.edu.cn

**Keywords:** shape memory alloys, finite element modeling, ratcheting, self-loosening of bolt

## Abstract

A macroscopic constitutive model is proposed in this research to reproduce the uniaxial transition ratcheting behaviors of the superelastic shape memory alloy (SMA) undergoing cyclic loading, based on the cosine-type phase transition equation with the initial martensite evolution coefficient that provides the predictive residual martensite accumulation evolution and the nonlinear feature of hysteresis loop. The calculated results are compared with the experimental results to show the validity of the present computational procedure in transition ratcheting. Finite element implementation for the self-loosening behavior of the superelastic SMA bolt is then carried out based on the proposed constitutive model to analyze the curves of stress-strain responses on the bolt bar, clamping force reduction law, dissipation energy change law of the bolted joint for different external loading cases, and preload force of the bolt.

## 1. Introduction

The fasteners, such as the bolt and lock washer, are used commonly in many types of structures. It has been evaluated that a large percentage of all mechanical failures are associated with the fastened joint failure [1]. Two modes are responsible for those joint failures: fatigue and self-loosening. The self-loosening of fasteners has been widely observed, whereby one becomes loose under dynamic loads in the form of transverse cyclic loading, shock, or vibration. Such failures can be catastrophic in safety-critical applications [2,3,4]. 

Recently, researchers have tried to explore the applications of shape memory alloy (SMA) materials, providing high damping, durability, and fatigue resistance, in the design of bolted joints, such as a SMA washer used to recover the initial preload in bolted joints [5], which produces a “self-healing bolted joint”, by combining piezoelectric-based health-monitoring techniques with SMA actuators to restore tension in a loose bolted joint. Another application of SMA washers as an actuator to increase the preload on loosened bolted joints was investigated with a novel model [6], based on a mathematical model developed by Ghorashi [7], by eliminating the preload term related to nut turning, making the system more practical. In these studies, the SMA actuator to recover the preload of the bolted joint was always designed as being the washer, for which it is relatively simpler to construct its stiffness model. However, the washer in a bolted joint can only bear the compression load and cannot take the bearing of the tension load. 

The other applications of SMA materials are the uses as a superelastic damper for civil engineering. The initial study of NiTi shape memory alloys’ behavior for use in structural and seismic application began in 1991, owing to their capability to provide supplemental damping to a structure, reducing inelastic behavior as subjected to cyclic loadings [8]. After then, both NiTi and Cu-based shape memory alloys were suggested for use as dampers for civil engineering applications based on studies of the material’s cyclic behavior [9,10,11]. After the brittle fractures of a large number of the steel moment-resisting frames with fully restrained welded beam-to-column connections found in the Northbridge earthquake in 1994 and later in the Kobe earthquake in 1995 [12,13], the SMA bolt was then introduced into the beam-to-column structure in T-stub connections because of the superelasticity. It was found that the T-stub connection with the SMA bolt presented all of the merits of bolted connections compared with the conventional high-strength bolt, but also contained a recentering capacity due to the lack of permanent residual deformation [14,15,16,17,18,19,20,21,22,23,24,25,26].

These studies focused on the different combinations of SMA and steel tendons tested to evaluate the recentering capability and the energy dissipative performance, not only for the hysteresis loop of single-cycle loading, but for the case being subjected to cyclic loadings. Some cases exhibit moderate energy dissipation and excellent recentering capability after cyclic loadings. The energy dissipation and ductility could be achieved under the deformation of the SMA bolts. These structure designs could effectively reserve the structural members (e.g., beam, column, and end-plate) within the elastic stage and mitigate the postearthquake repair work, thus considerably reducing the cost of reconstruction work.

However, these studies lack the capture of more subtle information of the relationship between the dissipated energy of the SMA bolted joint and the stiffness of those joint subparts. The stiffness of the SMA bolt and members directly influence the stress-strain relationship of the SMA bolt, and further determines the self-loosening of the bolted joint and its dissipated energy that is calculated by the area integration of the stress-strain curve of the SMA bolt. The preload force of the bolted joint would decrease with the cycle loading that is influenced by the phase transition law of the SMA bolt, and is reflected by the evolution of residual martensite deformation and phase transitions stress. 

The cyclic deformation behavior of SMAs, as described in earlier research, was followed with interested by the research group led by Miyazaki [27,28]. The group’s research shows that, under the action of mechanical cyclic loading, the residual plastic deformation of NiTi alloy would increase gradually with the increase of cycle times, and the phase transition stress would also decrease with the increase of cycle times. Strnadel [29,30] had studied the effect of alloy elements and components on the cyclic deformation behavior of superelastic NiTi alloy by experiments, and revealed the inhibition effect of high nickel content on cyclic residual deformation. At the same time, the effect of mechanical cyclic deformation on superelastic properties of alloys had been discussed. The deformation characteristics of superelastic NiTi alloy under cyclic loading and unloading were experimentally observed by Nemat-Nasser [31]. It was revealed that the increment of residual strain and the dissipation energy decrease with the increase of cycle times and tend to be stable after a certain number of cycles. 

Kang [32], using superelastic round bar NiTi alloy specimens, carried out the experimental study on the cyclic deformation characteristics of NiTi alloy under cyclic tension-unloading, cyclic tension-tension, and cyclic tension compression (symmetric and asymmetric). At the same time, the influence of different loading levels on the cyclic deformation behavior was discussed in that research.

As for the SMA bolted joint, the phase translation in the bolt bar would influence the stiffness of the SMA bolted joint, especially under a large external load. In this case, the self-loosening of the SMA bolt is not only determined by the material properties, but associated with the member stiffness of the SMA bolted joint. This paper thus explores the role of the external load and preload of the SMA bolted joint, as well as member stiffness on the self-loosening evolution of the SMA bolted joint. In addition, the dissipative energy of the SMA bolt is calculated for the load cases, and its relationship with the accumulated residual martensite strain of the SMA bolt during loading cycles is also examined. 

## 2. Constitutive Modeling for NiTi SMA under Cyclic Loading

In the last decade, some constitutive models were established to describe the cyclic deformation of the NiTi shape memory alloy. Compared with the micromechanics-based constitutive models of NiTi shape memory alloys, the phenomenological constitutive model is a good candidate for integrating into the structure computational methods [33], such as the finite element method, to predict the cyclic deformation of the NiTi alloy structure. However, these models with finite element implementation seem to make it difficult to efficiently analyze the complex structure, due to relatively low computation efficiency and nonlinearity.

Therefore, in this work, a simplified finite element model of the one-dimensional bar element is established to describe the elastic deformation of the bolt head and nut and the superelastic deformation with phase transition ratcheting of the SMA bolt, combining with the stiffness model of washers and members, and thus to further analyze the self-loosening of the SMA bolt under cyclic loadings. A one-dimensional constitutive model for the phase transition ratcheting of superelastic SMA is proposed based on the Brinson model, and then implemented into the finite element model to analyze the self-loosening of the SMA bolt. 

### 2.1. Constitutive Equation and Internal Variables

In the proposed model, the total strain ε can be decomposed into an elastic strain tensor $\varepsilon^{e}$ and an inelastic strain tensor with infinitesimal strain assumption.

(1) $$\varepsilon =\varepsilon^{e}+\varepsilon^{in}$$

The residual deformation of SMAs under cyclic loadings is attributed to the residual martensite deformation, due to the phase transition ratcheting and the accumulative martensite plastic deformation. In order to characterize these two deformation mechanisms, there are two internal variables that are introduced here in Helmoltz free energy, which is assumed to be additively decomposed into elastic and inelastic parts, as follows:

(2) $$\psi =\psi_{e}(\varepsilon -\varepsilon^{in},\xi ,\delta ,T)+\psi_{in}(\xi ,\delta ,T),$$

The internal variable ξ, selected as the martensite volume fraction, is to depict the stress-induced martensite phase transition, and is constrained by 0 ≤ ξ ≤ 1. δ characterizes the accumulative martensite transition, including the accumulated martensite volume fraction $\delta_{c}$. T is the temperature. Based on the assumption that the elastic thermal effect is negligible, the elastic free energy function can be chosen as:

(3) $$\psi_{e}=\sigma (\varepsilon -\varepsilon^{in}),$$

Then, the stress and, accordingly, the state equation can be derived as:

(4) $$\sigma =\frac{\partial \psi }{\partial (\varepsilon -\varepsilon^{in})}=D\left(\xi \right)(\varepsilon -\varepsilon^{in}),$$

in which σ and D are the stress tensor and the elastic modulus matrix, respectively. Those are the functions of the martensite volume fraction ξ.

From the principle of thermodynamics, Clausius-Duhem inequality can be expressed as

(5) $$\sigma \dot{\varepsilon }-{\dot{\psi }}_{e}(\varepsilon -\varepsilon^{in},\xi ,\delta ,T)-{\dot{\psi }}_{in}(\xi ,\delta ,T)\geq 0,$$

where

(6) $${\dot{\psi }}_{e}=\sigma (\dot{\varepsilon }-{\dot{\varepsilon }}^{in})=\frac{\partial \psi }{\partial (\varepsilon -\varepsilon^{in})}(\dot{\varepsilon }-{\dot{\varepsilon }}^{in}),$$

(7) $$\psi_{in}=\frac{\partial \psi }{\partial \xi }\dot{\xi }+\frac{\partial \psi }{\partial \delta }\dot{\delta }=\frac{\partial \psi }{\partial \xi }\dot{\xi }+\frac{\partial \psi }{\partial \delta_{c}}{\dot{\delta }}_{c}+\frac{\partial \psi }{\partial \delta_{p}}{\dot{\delta }}_{p},$$

According to the description in [32,33,34], an incomplete reverse transition from stress-induced martensite to austenite could be observed during the cyclic phase transition. Furthermore, the amount of residual martensite would increase with the increasing number of cycles during the cyclic loadings. Therefore, the total induced-martensite volume fraction ξ that represents the progressive increase of residual strain should be divided into two parts, i.e., the reversible martensite volume fraction, $\xi_{r}$, and the irreversible residual one, $\xi_{ir}$.

(8) $$\xi =\xi_{r}+\xi_{ir}$$

From the experimental results, the phase transition deformation evolves with the increasing number of loading cycles and reaches stability after a certain number of cycles [32,33,34]. In order to take these evolution processes into account, the internal variable $\delta_{c}$ is selected as the accumulated martensite volume fraction $\xi_{c}$ that represents the evolution process of some variables and material parameters with the increasing number of cycles, and is written by

(9) $$\xi_{c}=\int_{0}^{t}\left|{\dot{\xi }}_{r}(\tau )\right|d\tau$$

where *t* is a kinematic time point. 

It should be noted that in the studies by Kang [33] and Lagoudas [35], the accumulated martensite volume fraction depends on the martensite volume fraction ξ that is the superposition of the reversible martensite volume fraction $\xi_{r}$ and the irreversible residual one $\xi_{ir}$. However, in this research, the irreversible martensite volume fraction can be defined as the function of the reversible martensite volume fraction. Therefore, in order to simplify the derivation process of the incremental formulation of constitutive equations that will be discussed in the following sections, the accumulated martensite volume fraction is considered to be dependent on the reversible martensite volume fraction instead of the irreversible martensite volume fraction.

Based on the general plasticity, the total inelastic strain $\varepsilon^{in}$ in Equation (1) is equal to the transition strain $\varepsilon^{tr}$ from the stress-induced martensite phase and its reverse.

(10) $$\varepsilon^{in}=\varepsilon^{tr}$$

The SMA constitutive model used here is based on a model originally formulated by [36,37,38], which is a phenomenological macroscale one-dimensional constitutive model and can be written as

(11) $$\sigma -\sigma_{0}=E^{s}(\xi )\varepsilon -E^{s}(\xi_{0})\varepsilon_{0}+\Theta (T-T_{0})+\Omega (\xi )\xi -\Omega (\xi_{0})\xi_{0}$$

where $E^{s}$ is the Young’s modulus, Ω is the transition coefficient, and Θ is the thermal elastic coefficient. The subscript ‘0’ indicates the initial values. Young’s modulus $E^{s}$ and the transition coefficient Ω are the functions of the martensite volume fraction ξ, which are given as

(12a) $$E^{s}(\xi )=E_{A}+\xi (E_{M}-E_{A})$$

(12b) $$\Omega (\xi )=-\varepsilon_{L}E^{s}(\xi )$$

in which $\varepsilon_{L}$ is the maximum transition strain obtained from the uniaxial tension test.

It is assumed that the equivalent transition strain and the recoverable martensite volume fraction show a proportional relationship (irreversible martensitic transition does not participate in the transition process). Then, the following relationship can be obtained:

(13) $$\xi =\frac{\varepsilon^{tr}}{\varepsilon_{L}}.$$

Equation (8) depicts that the total induced-martensite volume fraction ξ consists of the reversible martensite volume fraction $\xi_{r}$ and the irreversible residual one $\xi_{ir}$. Since the irreversible martensitic transition does not take part in the phase transition process, the stress is only the function of the reversible martensite volume fraction, which is irrelevant to the residual martensite volume fraction in the phase transition. The expression forms of $\xi_{r}$ are as follows:

(i) Transition to the martensite phase
  if $T>M_{S}$ and $\sigma_{s}^{cr}+C_{M}(T-M_{s})<\sigma <\sigma_{f}^{cr}+C_{M}(T-M_{s})$:
  (14) $$\underset{A\to M}{\xi_{r}}=\frac{1-\underset{M\to A}{\xi_{ir}^{f}}}{2}\cos\left\{\frac{\pi }{\sigma_{s}^{cr}-\sigma_{f}^{cr}}[\sigma -\sigma_{f}^{cr}-C_{M}(T-M_{S})]\right\}+\frac{1+\underset{M\to A}{\xi_{ir}^{f}}}{2}$$
(ii) Transition to the austenite phase
  if $T>A_{S}$ and $C_{A}(T-A_{f})<\sigma <C_{A}(T-A_{s})$:
  (15) $$\underset{M\to A}{\xi_{r}\;}=\frac{\underset{A\to M}{\xi_{r}^{f}}-\underset{M\to A}{\xi_{ir}}}{2}\left\{\cos[a_{A}(T-A_{s}-\frac{\sigma }{C_{A}})]+1\right\}$$
  where $\sigma_{s}^{cr}$ and $\sigma_{f}^{cr}$ are the starting and finishing stresses of martensite transition, respectively; $C_{A}$ and $C_{M}$ are the slopes for the relation between critical transition stress and temperature, respectively; $A_{s}$ and $A_{f}$ are the starting and finishing temperatures of austenite transition, respectively; $M_{s}$ and $M_{f}$ are the starting and finishing temperatures of martensite transition, respectively; $\underset{M\to A}{\xi_{ir}^{f}}$ is the residual volume fraction of martensite transition at the end of transition to the austenite phase; and $\underset{A\to M}{\xi_{r}^{f}}$ is the volume fraction of the martensite transition at the end of transition to the martensite phase.

It should be noted that the proposed model is the extension of Brinson’s work [36]. The difference is in the cosine-type phase transformation equation for the expression of $\xi_{r}$. In this work, an initial martensite evolution coefficient of the cosine-type phase transformation equation is introduced to describe the phase transformation behaviors of the SMA undergoing cyclic loading to predict the residual martensite accumulation. 

It is assumed in the proposed model that the martensite accumulation only happens in the process of reverse transformation. Based on this assumption, the initial evolution coefficient $\left(1-\underset{M\to A}{\xi_{ir}^{f}}\right)/2$ of the cosine-type function in Equation (14) is constant in the forward transformation, and implies the austenite content as the applied loading returns to zero or the maximum austenite content that is able to transfer to the martensite phase in the forward transformation. On the other hand, the irrecoverable martensite volume fraction $\underset{M\to A}{\xi_{ir}}$ is changeable in real time in the reverse transformation, which is a function of stress and will be discussed in the next section in detail. It implies that the initial evolution coefficient $\left(\underset{A\to M}{\xi_{r}^{f}}-\underset{M\to A}{\xi_{ir}}\right)/2$ of the cosine-type function in Equation (15) that will affect transformation start conditions is a real-time variable in the reverse transformation.

The parameter $a_{A}$ is expressed as

(16) $$a_{A}=\frac{\pi }{A_{f}-A_{s}}$$

Now, Equation (11) can be rewritten considering the irrecoverable feature as follows:

(17) $$\sigma -\sigma_{0}=E^{s}(\xi )\varepsilon -E^{s}(\xi_{0})\varepsilon_{0}+\Theta (T-T_{0})+\Omega (\xi )\xi_{r}-\Omega (\xi_{0})\xi_{0}$$

The research by Kan [33] had discussed the tension-compression responses under cyclic loading. However, the experiments by Kang [32] had showed that the transformation ratcheting has asymmetry. A simple Drucker-Prager-type equivalent stress was introduced in Kan-Kang’s model [33] to consider the difference between tensile and compressive behaviour. In this research, although the proposed model could describe the tension-compression stress responses, it is not necessary to consider the tension-compression asymmetry owing to the fact that the stress of the bolt is always under the status of being tensile.

### 2.2. Evolution Law of Parameters Governed by Accumulated Martensite Volume Fraction 

From the descriptions in [32,33,34,35,39], the transition ratcheting and transition stresses evolving with the cycle numbers are related to the accumulated martensite volume fraction $\delta_{c}$. It was concluded by Lagoudas [35] that the evolution of peak strain and valley strain was attributed to the transition ratcheting, and provided an evolution equation of the transition ratcheting by taking the accumulated martensite volume fraction as the governing variable. However, the further research by Kan [33] found that the parameters of transition ratcheting and transition stress associated with the loading stress level. The detailed evolution equations can be referred to in the previous work [33]. For the integrity of the content in this work, the evolution equations are outlined as follows:

(i) Evolution equation for the residual martensite volume fraction:
  (18) $${\dot{\xi }}_{ir}=\xi_{ir\max}c_{AM}^{f}(\sigma )e^{-b\xi_{c}}{\dot{\xi }}_{c},$$
  where $\xi_{ir\max}$ is the maximum irreversible residual martensite volume fraction corresponding to the maximum load of the stable cycle phase transition. The material parameter *b* is to govern the saturation rate of the residual martensite volume fraction $\xi_{ir}$. The revised function $c_{MA}(\sigma )$ is introduced in the evolution law and associated with the loading stress level to consider the correlation between that level and the residual martensite fraction, and written as
  (19a) $$c_{AM}^{}(\sigma )={\left(\frac{<Q_{f}^{AM}-Q_{s}^{AM}-<Q_{f}^{AM}-\sigma >>}{Q_{f}^{AM}-Q_{s}^{AM}}\right)}^{n}$$
  (19b) $$c_{AM}^{f}(\sigma )=\max(c_{AM}^{f}(\sigma ))$$
  where $Q_{f}^{AM}=\sigma_{f}^{AM}$, $<x>=\frac{1}{2}(x+|x|)$, and $c_{AM}^{f}$ is the value of $c_{AM}^{}$ at the endpoint of the forward transition. The material parameter *n* is to describe the nonlinear relationship between the residual martensite volume fraction and the loading stress level. It can be found according to Equation 19a that in the forward phase, the value of $c_{AM}^{}$ increases with the increase of the loading stress level in the interval of $Q_{s}^{AM}\leq \sigma \leq Q_{f}^{AM}$, and reaches its maximum at the endpoint of the forward transition.
(ii) Evolution law of transition stress
  Due to incomplete reverse transition during loading cycles, the superelastic NiTi SMA shows the mixture state of the austenite and residual martensite phases. The transition stresses decrease with the increasing of cycle numbers. Thus, according to the experimental observations in [32], the evolution equations in exponential formulation were proposed by [33] to describe the progressive evolution of the transition stresses with increasing cycle numbers from their initial values to stable ones, and are introduced here as
  (20a) $$\sigma_{s}^{AM}=\sigma_{s_{0}}^{AM}-(\sigma_{s_{0}}^{AM}-\sigma_{s_{1}}^{AM})(1-e^{-c_{s}^{AM}\xi_{c}}),$$
  (20b) $$\sigma_{f}^{AM}=\sigma_{f_{0}}^{AM}-(\sigma_{f_{0}}^{AM}-\sigma_{f_{1}}^{AM})(1-e^{-c_{f}^{MA}\xi_{c}}),$$
  (20c) $$\sigma_{s}^{MA}=\sigma_{s_{0}}^{MA}-(\sigma_{s_{0}}^{MA}-\sigma_{s_{1}}^{MA})(1-e^{-c_{s}^{MA}\xi_{c}}),$$
  (20d) $$\sigma_{f}^{MA}=\sigma_{f_{0}}^{MA}-(\sigma_{f_{0}}^{MA}-\sigma_{f_{1}}^{MA})(1-e^{-c_{f}^{MA}\xi_{c}}),$$
  where $\sigma_{s_{0}}^{AM}$, $\sigma_{f_{0}}^{AM}$, $\sigma_{s_{0}}^{MA}$, and $\sigma_{f_{0}}^{MA}$ are the transition stresses of the initial cyclic loading, and $\sigma_{s_{1}}^{AM}$, $\sigma_{f_{1}}^{AM}$, $\sigma_{s_{1}}^{MA}$, and $\sigma_{f_{1}}^{MA}$ are the transition stresses of the stable phase transition. $c_{s}^{AM}$, $c_{f}^{AM}$, $c_{s}^{MA}$, and $c_{f}^{MA}$ are the parameters governing the saturated rates of the transition stresses.

### 2.3. Incremental Formulation of Constitutive Equations

Based on the infinitesimal strain assumption, the increment of total strain dε can be defined as the summation of the increments of elastic strain and transition strain.

(21) $$d\varepsilon =d\varepsilon^{e}+d\varepsilon^{tr}$$

Furthermore, based on Equation (17), the stress increment can be calculated using the following equation:

(22) $$\Delta \sigma =\varepsilon \frac{dE^{s}}{d\xi }\frac{d\xi }{d\sigma }\Delta \sigma +E^{s}\Delta \varepsilon +\left(\xi_{r}\frac{d\Omega }{dE^{s}}\frac{dE^{s}}{d\xi }\frac{d\xi }{d\sigma }+\Omega \frac{d\xi_{r}}{d\sigma }\right)\Delta \sigma$$

Derived from Equation (12), the incremental formulation of some variables in Equation (22) can be obtained as

(23a) $$\frac{dE^{s}}{d\xi }=E_{M}-E_{A},$$

(23b) $$\frac{d\Omega }{dE^{s}}=-\varepsilon_{L},$$

(23c) $$\Delta \xi_{c}=\Delta \xi_{r}=\frac{d\xi_{r}}{d\sigma }\Delta \sigma .$$

Combining with Equation (23), the incremental stress can be rewritten as

(24) $$\Delta \sigma =\varepsilon \left(E_{m}-E_{a}\;\right)\frac{d\xi }{d\sigma }\Delta \sigma +E^{s}\Delta \varepsilon +\left[\Omega \frac{d\xi_{r}}{d\sigma }-\xi_{r}\varepsilon_{L}\left(E_{m}-E_{a}\right)\frac{d\xi }{d\sigma }\right]\Delta \sigma$$

The incremental stress-strain relationship is further expressed as follows:

(25a) $$D^{sma}=\frac{\beta }{\alpha },$$

(25b) $$\alpha =[1-\varepsilon \left(E_{m}-E_{a}\right)\frac{d\xi }{d\sigma }+c_{p}d_{AM}(\sigma )e^{-d_{p}\xi_{c}}E^{s}\frac{d\xi_{r}}{d\sigma }-\Omega \frac{d\xi_{r}}{d\sigma }+\xi_{r}\varepsilon_{L}\left(E_{m}-E_{a}\right)\frac{d\xi }{d\sigma }],$$

(25c) $$\beta =E^{s}.$$

Now, $D^{sma}$ cannot be obtained yet, unless the increments of the phase transition $d\xi_{r}/d\sigma$ and dξ/dσ are given. The derivation process of the increments of the phase transition will be discussed as follows.

(i) Transition to the martensite phase

According to the model discussed in [36], the forward transition stresses can be expressed as

(26a) $$\sigma_{s}^{AM}=\sigma_{s}^{cr}+C_{M}(T-M_{s}),$$

(26b) $$\sigma_{f}^{AM}=\sigma_{f}^{cr}+C_{M}(T-M_{s}).$$

Therefore, Equation (14) is rewritten as

(27) $$\underset{A\to M}{\xi_{r}}=\frac{1-\underset{M\to A}{\xi_{ir}^{f}}}{2}\cos\left\{\frac{\pi }{\left(\sigma_{s}^{AM}-\sigma_{f}^{AM}\right)}[\sigma -\sigma_{f}^{AM}]\right\}+\frac{1+\underset{M\to A}{\xi_{ir}^{f}}}{2}$$

The incremental formulation of Equation (27) can be further expressed as

(28) $$\frac{\underset{A\to M}{d\xi_{r}}}{d\sigma }=\Gamma_{0}^{AM}\frac{d\left\{\left[\sigma -\sigma_{f}^{AM}\right]/\left(\sigma_{s}^{AM}-\sigma_{f}^{AM}\right)\right\}}{d\sigma },$$

where

(29) $$\Gamma_{0}^{AM}=-\frac{\pi \left(1-\underset{M\to A}{\xi_{ir}^{f}}\right)}{2}\sin\{\frac{\pi }{\left(\sigma_{s}^{AM}-\sigma_{f}^{AM}\right)}[\sigma -\sigma_{f}^{AM}]\};$$

and

(30) $$\frac{d\left\{\left[\sigma -\sigma_{f}^{AM}\right]/\left(\sigma_{s}^{AM}-\sigma_{f}^{AM}\right)\right\}}{d\sigma }=\frac{1}{\sigma_{s}^{AM}-\sigma_{f}^{AM}}+\Gamma_{1}^{AM}\frac{\underset{A\to M}{d\xi_{r}}}{d\sigma }+\Gamma_{2}^{AM}\frac{\underset{A\to M}{d\xi_{r}}}{d\sigma },$$

where

(31a) $$\Gamma_{1}^{AM}=\frac{ce^{-c\xi_{c}}(\sigma_{f0}^{AM}-\sigma_{f1}^{AM})}{\sigma_{s}^{AM}-\sigma_{f}^{AM}},$$

(31b) $$\Gamma_{2}^{AM}=-\frac{ce^{-c\xi_{c}}\left[\left(\sigma_{f_{0}}^{AM}-\sigma_{f_{1}}^{AM}\right)-\left(\sigma_{s_{0}}^{AM}-\sigma_{s_{1}}^{AM}\right)\right]\left[\sigma -\sigma_{f}^{AM}\right]}{{\left(\sigma_{s}^{AM}-\sigma_{f}^{AM}\right)}^{2}}.$$

Substituting Equation (30) into Equation (28), the following equation can be derived as

(32) $$\frac{\underset{A\to M}{d\xi_{r}}}{d\sigma }=\Gamma_{0}^{AM}(\frac{1}{\sigma_{s}^{AM}-\sigma_{f}^{AM}}+\Gamma_{1}^{AM}\frac{\underset{A\to M}{d\xi_{r}}}{d\sigma }+\Gamma_{2}^{AM}\frac{\underset{A\to M}{d\xi_{r}}}{d\sigma }),$$

and further obtained thus

(33) $$\frac{\underset{A\to M}{d\xi_{r}}}{d\sigma }=\frac{\Gamma_{0}^{AM}}{\left(\sigma_{s}^{AM}-\sigma_{f}^{AM}\right)\left(1-\Gamma_{0}^{AM}\Gamma_{1}^{AM}-\Gamma_{0}^{AM}\Gamma_{2}^{AM}\right)},$$

(ii) Transition to the austenite phase

For the reverse transition, the incremental formulation of Equation (15) can be written as

(34) $$\frac{\underset{M\to A}{d\xi_{r}}}{d\sigma }=\Gamma_{0}^{MA}\frac{d[a_{A}(T-A_{s}-\sigma /C_{A})]}{d\sigma }-\frac{1}{2}\left\{\cos[a_{A}(T-A_{s}-\frac{\sigma }{C_{A}})]+1\right\}\frac{\underset{M\to A}{d\xi_{ir}}}{d\sigma },$$

where

(35) $$\Gamma_{0}^{MA}=-\frac{\underset{A\to M}{\xi_{r}^{f}}-\underset{M\to A}{\xi_{ir}}}{2}\sin[a_{A}(T-A_{s}-\frac{\sigma }{C_{A}})].$$

According to Equation (18), it can be given

(36) $$\frac{\underset{M\to A}{d\xi_{ir}}}{d\sigma }=\xi_{ir\max}c_{AM}^{f}be^{-b\xi_{c}}\frac{d\xi_{c}}{d\sigma }=\xi_{ir\max}c_{AM}^{f}be^{-b\xi_{c}}\frac{\underset{M\to A}{d\xi_{r}}}{d\sigma },$$

With the description in [36], the reverse transition stresses can be expressed as

(37a) $$\sigma_{s}^{MA}=C_{A}(T-A_{s}),$$

(37b) $$\sigma_{f}^{MA}=C_{A}(T-A_{f}).$$

Another expression can be written as

(38a) $$A_{s}=T-\sigma_{s}^{MA}/C_{A},$$

(38b) $$A_{f}=T-\sigma_{f}^{MA}/C_{A}.$$

Therefore, Equation (16) can be expressed as

(39) $$a_{A}=\frac{\pi }{A_{f}-A_{s}\;}=\frac{\pi C_{A}}{\sigma_{s}^{MA}-\sigma_{f}^{MA}}$$

Thus,

(40) $$\begin{array}{c} \frac{d\left\{a_{A}\left[T-A_{s}-\sigma /C_{A}\right]\right\}}{d\sigma }=\frac{d\left\{\pi \left[\sigma_{s}^{MA}-\sigma \right]/\left(\sigma_{s}^{MA}-\sigma_{f}^{MA}\right)\right\}}{d\sigma }\\ =\Gamma_{1}^{MA}\frac{\underset{M\to A}{d\xi_{r}}}{d\sigma }+\Gamma_{2}^{MA}\frac{\underset{M\to A}{d\xi_{r}}}{d\sigma }-\frac{\pi }{\left(\sigma_{f}^{MA}-\sigma_{s}^{MA}\right)}, \end{array}$$

where

(41a) $$\Gamma_{1}^{MA}=\frac{-\pi ce^{-c\xi_{c}}(\sigma_{s_{0}}^{MA}-\sigma_{s_{1}}^{MA})}{\sigma_{s}^{MA}-\sigma_{f}^{MA},}$$

(41b) $$\Gamma_{2}^{MA}=\frac{\pi ce^{-c\xi_{c}}\left[\sigma_{s}^{MA}-\sigma /C_{A}\right]\left[\left(\sigma_{s_{0}}^{MA}-\sigma_{s_{1}}^{MA})-(\sigma_{f_{0}}^{MA}-\sigma_{f_{1}}^{MA}\right)\right]}{{\left(\sigma_{s}^{MA}-\sigma_{f}^{MA}\right)}^{2}}.$$

Substituting Equations (36) and 40) into Equation (34), it can be derived as

(42) $$\frac{\underset{M\to A}{d\xi_{r}}}{d\sigma }=\left\{ \begin{array}{l} \frac{\pi \Gamma_{0}^{MA}}{\left(\sigma_{s}^{MA}-\sigma_{f}^{MA}\right)\left(\Gamma_{0}^{MA}\Gamma_{1}^{MA}+\Gamma_{0}^{MA}\Gamma_{2}^{MA}+\Gamma_{3}^{MA}-1\right)}\begin{array}{ccccc} & & & & \sigma \geq 0 \end{array}\\ \frac{\pi \left(\alpha -1\right)\Gamma_{0}^{MA}}{\left(1+\alpha \right)\left(\sigma_{s}^{MA}-\sigma_{f}^{MA}\right)\left(\Gamma_{0}^{MA}\Gamma_{1}^{MA}+\Gamma_{0}^{MA}\Gamma_{2}^{MA}+\Gamma_{3}^{MA}-1\right)}\begin{array}{ccc} & & \sigma <0 \end{array} \end{array}\right.,$$

where

(43) $$\Gamma_{3}^{MA}=-\frac{1}{2}\xi_{ir\max}c_{AM}^{f}be^{-b\xi_{c}}\{\cos[a_{A}(T-A_{s}-\frac{\sigma }{C_{A}})]+1\}.$$

Thus, the incremental formulation of the martensite volume fraction ξ can be derived via Equation (9):

(44) $$\frac{d\xi }{d\sigma }=\frac{d\xi_{r}}{d\sigma }+\frac{d\xi_{ir}}{d\sigma },$$

and further obtained thus

(45) $$\left\{ \begin{array}{l} \frac{\underset{A\to M}{d\xi }}{d\sigma }=\frac{\underset{A\to M}{d\xi_{r}}}{d\sigma }\\ \frac{\underset{M\to A}{d\xi }}{d\sigma }=\frac{\underset{M\to A}{d\xi_{r}}}{d\sigma }+\frac{\underset{M\to A}{d\xi_{ir}}}{d\sigma }=\left(1+\xi_{ir\max}c_{AM}^{f}be^{-b\xi_{c}}\right)\frac{\underset{M\to A}{d\xi_{r}}}{d\sigma } \end{array}.\right.$$

Finally, substituting Equations (33), (41) and (45) into Equation (25), the incremental stress-strain relationship can be obtained. 

### 2.4. Finite Element Modeling for the One-Dimensional Bar Element of SMA Ratcheting Behavior 

Some scholars had put forward an improved finite element method based on the traditional finite element method, that is, the method of consecutive interpolation. Nguyen et al. developed a novel enhanced eight-node hexahedral element to analysis three-dimensional liner solids and composite structures. Another formulation is a novel consecutive interpolation of a four-node tetrahedral finite element to analysis the heat transfer problems in three-dimensional models [40,41]. In order to construct the finite element model of the one-dimensional bar element of superelastic NiTi SMA ratcheting behavior, the constitutive equation as Equation (17) should be expressed as the incremental formulation. It should be noted that the incremental equation in the proposed finite element model can provide an explicit expression of the incremental relationship of the stress and strain that can ensure the convergence of the numerical calculation. The increment stiffness matrix of the bar element should be further derived. Therefore, Equation (17) needs first to be expressed as the relationship of the change in nodal force and the elemental deformation in length. Then, it is carried through a resolution of the nonlinear equation by the variational method to obtain the stiffness matrix. The relationship between force and deformation is expressed as

(46) $$F^{s}-F_{0}^{s}=E^{s}(\xi )A^{s}\ln\left(\frac{L^{s}}{L_{0}^{s}}\right)\frac{x}{L^{s}}+\left[\Omega (\xi )\xi_{r}A^{s}-E(\xi_{0})\varepsilon_{0}A^{s}-\Omega (\xi_{0})\xi_{0}A^{s}\right]\frac{x}{L^{s}},$$

where $F^{s}=\sigma^{s}A^{s}\frac{x}{L^{s}}$ is the force vector of the bar element, $F_{0}^{s}$ is the initial force vector of the bar element, $\sigma^{s}$ is the axial stress of the SMA bolt bar, $A^{s}$ is the cross-sectional area of the bar element, $L_{0}^{s}$ is the unstressed initial length of the bar element, $L^{s}$ is the real-time length of the bar element after deformation, and $x/L^{s}$ is the nodal position vectors of the bar element. Thus, for Equation (46), $n_{AM}=n_{MA}=x/L^{s}$.

Since the phase transition strain is a relatively large value, the total strain is calculated in the logarithmic formulation $\varepsilon =\ln\left(L^{s}/L_{0}^{s}\right)$ to approximate the real one. 

We set

(47) $$Q=\Omega \xi_{r}-E_{0}^{s}\varepsilon_{0}-\Omega_{0}\xi_{0},$$

and hence, Equation (46) can be simplified as

(48) $$F^{s}=E^{s}A^{s}\ln\left(\frac{L^{s}\;}{L_{0}^{s}}\right)\frac{x}{L^{s}}+Q\frac{A^{s}x}{L^{s}}+F_{0}^{s}$$

Combining the equations,

(49) $$\left\{ \begin{array}{l} \Delta \sigma =\frac{x_{}^{T}}{A^{s}L^{s}}\Delta F^{s}\\ \Delta \left(\frac{x}{L^{s}}\right)=\left(\frac{1}{L^{s}}I_{3\times 3}-\frac{xx_{}^{T}}{L^{s}{}^{3}}\right)\Delta x\\ \Delta E^{s}=\frac{dE^{s}}{d\xi }\frac{d\xi }{d\sigma }\Delta \sigma =\frac{dE^{s}}{d\xi }\frac{d\xi }{d\sigma }\frac{x_{}^{T}}{A^{s}L^{s}}\Delta F^{s}\\ \Delta \left[\ln\left(\frac{L^{s}}{L_{0}^{s}}\right)\frac{x}{L^{s}}\right]=\left[\frac{1}{L^{s}}\frac{x_{}^{T}x}{L^{s}{}^{2}}+\ln\left(\frac{L^{s}}{L_{0}^{s}}\right)\left(\frac{1}{L^{s}}I_{3\times 3}-\frac{xx_{}^{T}}{L^{s}{}^{3}}\right)\right]\Delta x=\left\{\left[1+\ln\left(\frac{L^{s}}{L_{0}^{s}}\right)\right]\frac{1}{L^{s}}I_{3\times 3}-\ln\left(\frac{L^{s}}{L_{0}^{s}}\right)\frac{xx_{}^{T}}{L^{s}{}^{3}}\right\}\Delta x\\ \Delta Q=\Delta \left(\Omega \xi_{r}\right)=\left(\xi_{r}\frac{d\Omega }{dE^{s}}\frac{dE^{s}}{d\xi }\frac{d\xi }{d\sigma }+\Omega \frac{d\xi_{r}}{d\sigma }\right)\Delta \sigma =\left(\xi_{r}\frac{d\Omega }{dE^{s}}\frac{dE^{s}}{d\xi }\frac{d\xi }{d\sigma }+\Omega \frac{d\xi_{r}}{d\sigma }\right)\frac{x_{}^{T}}{A^{s}L^{s}}\Delta F^{s} \end{array}\right.$$

the variation of the formula of Equation (48) is

(50) $$\begin{array}{l} \Delta F^{s}=\ln\left(\frac{L^{s}}{L_{0}^{s}}\right)\frac{dE^{s}}{d\xi }\frac{d\xi }{d\sigma }\frac{xx_{}^{T}}{L^{s}{}^{2}}\Delta F^{s}+\left\{\left[1+\ln\left(\frac{L^{s}}{L_{0}^{s}}\right)\right]\frac{E^{s}A^{s}}{L^{s}}I_{3\times 3}-\ln\left(\frac{L^{s}}{L_{0}^{s}}\right)\frac{E^{s}A^{s}}{L^{s}}\frac{xx_{}^{T}}{L^{s}{}^{2}}\right\}\Delta x\\ +\left(\xi_{r}\frac{d\Omega }{dE^{s}}\frac{dE^{s}}{d\xi }\frac{d\xi }{d\sigma }+\Omega \frac{d\xi_{r}}{d\sigma }\right)\frac{xx_{}^{T}}{L^{s}{}^{2}}\Delta F^{s}+A^{s}Q\left(\frac{1}{L^{s}}I_{3\times 3}-\frac{xx_{}^{T}}{L^{s}{}^{3}}\right)\Delta x \end{array}.$$

It is rearranged further as

(51) $$\Delta F^{s}=\frac{\beta }{\alpha }\Delta x=K_{U}^{sma}\Delta x,$$

and

(52) $$K_{U}^{sma}=\frac{\beta }{\alpha },$$

which depicts the stiffness matrix of the single bar element. In the stage of phase transition, the variables of α and β are written as

$$\begin{array}{l} \alpha =I_{3\times 3}-\frac{dE^{s}}{d\xi }\frac{d\xi }{d\sigma }\left(\frac{xx_{}^{T}}{L_{0}^{s}L^{s}}-\frac{xx_{}^{T}}{L^{s}{}^{2}}\right)-\left(\xi_{r}\frac{d\Omega }{dE^{s}}\frac{dE^{s}}{d\xi }\frac{d\xi }{d\sigma \;}+\Omega \frac{d\xi_{r}}{d\sigma }\right)\frac{xx_{}^{T}}{L^{s}{}^{2}}+\left(\frac{L_{p}^{s}}{L_{0}^{s}}\frac{dE^{s}}{d\xi }\frac{d\xi }{d\sigma }+\Delta L_{p}^{s}\frac{d\xi_{r}}{d\sigma }\right)\frac{xx_{}^{T}}{L^{s}{}^{2}}\\ \beta =\left\{\left[1+\ln\left(\frac{L^{s}}{L_{0}^{s}}\right)\right]\frac{E^{s}A^{s}}{L^{s}}I_{3\times 3}-\ln\left(\frac{L^{s}}{L_{0}^{s}}\right)\frac{E^{s}A^{s}}{L^{s}}\frac{xx_{}^{T}}{L^{s}{}^{2}}\right\}+\left(A^{s}Q-A^{s}R\right)\left(\frac{1}{L^{s}}I_{3\times 3}-\frac{xx_{}^{T}}{L^{s}{}^{3}}\right) \end{array}$$

For the stage of non-phase transition, $\alpha =I_{3\times 3}$.

From Equation (63), it can be derived

(53) $$\left\{ \begin{array}{l} \frac{dE^{s}}{d\xi }=E_{m}-E_{a}\\ \frac{d\Omega }{dE^{s}}=-\varepsilon_{L} \end{array}.\right.$$

With these, the stiffness matrix $K_{U}^{sma}$ of the single bar element of a SMA can be obtained.

### 2.5. Numerical Simulation and Model Verification

**Figure 1:**
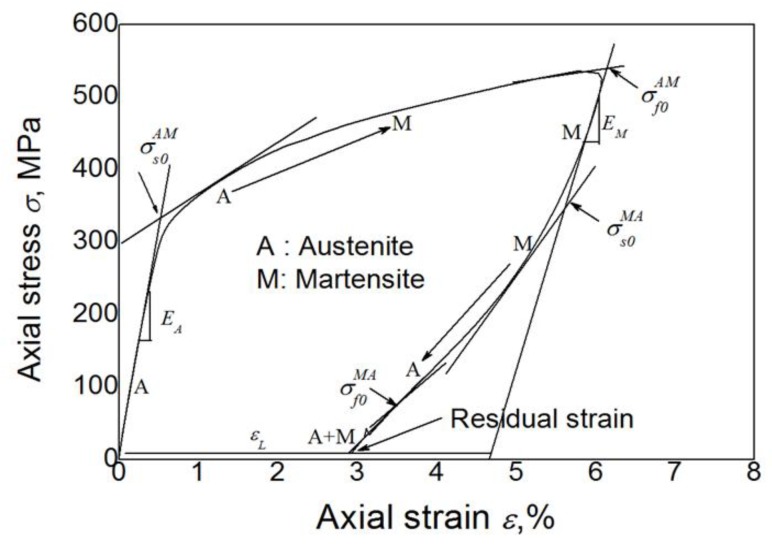
Typical tension-unloading stress-strain curve of materials [32].

**Figure 2:**
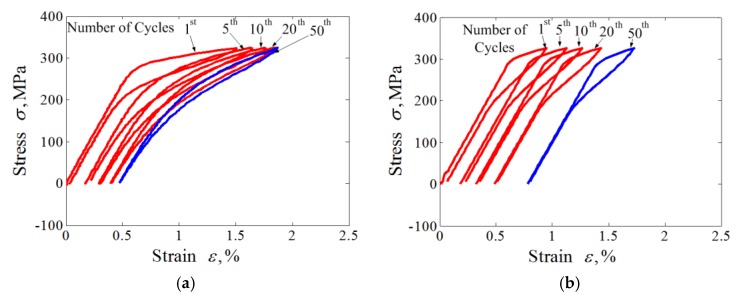

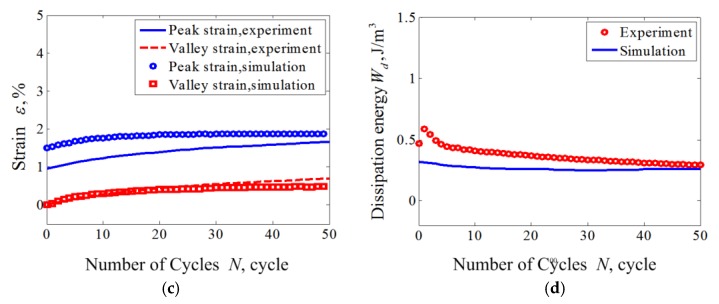
Experiments and simulations for cyclic tension-unloading transition ratchetting with constant loading stress of 325 MPa: (**a**) experimental results; (**b**) simulated results; (**c**) curves of peak and valley strains vs. cycle numbers; (**d**) curves of dissipation energy vs. cycle numbers.

In this section, cyclic responses for ratcheting associated with the one-dimensional cyclic behaviour of SMAs are verified by the uniaxial thermomechanical cyclic experiments obtained in the current work and referable literature [33], describing the transition ratcheting during the stress-controlled cyclic loading at room temperature. The typical tensile and unloading stress-strain curve of the superelastic NiTi SMA at room temperature is shown in Figure 1. It is shown from Figure 2 that the superelastic NiTi SMA manifests an apparently superelastic feature. However, its curve presents slightly differently from the description in the referred literature for the NiTi SMA manufactured by other companies (e.g., SMA, San Jose, CA, USA). It shows an apparent hardening during the stress-induced martensite transition. After unloading, it could be found that a relatively high residual strain (*ε_r_* = 1.5%) remained. The high residual strain implies that there is an incomplete reverse transition from the stress-induced martensite to the original austenite after unloading, which leads to some amount of martensite strain remaining. It is further found that the amount of remained martensite increases progressively with the cyclic loadings. This accumulation phenomenon of deformation had been named as “phase transition ratcheting” and discussed in detail by [26,27,28,29,30,31,32], which is different from the ratcheting of ordinary metals without phase transition. It illustrates that after the first unloading, the pure austenite is replaced by the mixture of austenite and remaining martensite in the alloy. The stresses are certainly no longer the transition stresses of the pure austenite and martensite. 

Prior to discussing the ratcheting deformation of the superelastic NiTi SMA, some parameters are defined as shown in Figure 1. The defined parameters contain the elastic modulus of austenite *E_A_* and martensite *E_M_*, the starting stress of the forward phase transition of austenite to martensite $\sigma_{s}^{AM}$ and the inverse transition of martensite to austenite $\sigma_{s}^{MA}$, the finishing stress of the forward phase transition of austenite to martensite $\sigma_{f}^{AM}$, and the inverse transition of martensite to austenite $\sigma_{f}^{MA}$. The subscript ‘0’ here indicates the first phase transition cycle of cyclic tension-unloading. It should be noted that the parameters of elastic modulus and transition stress are nominal variables due to the existence of residual martensite and its change during the cyclic loadings. Moreover, the dissipation energy *W_d_* is defined as the area around the stress-strain curve in each loading-unloading cycle:

(54) $$W_{d}=\oint \sigma d\varepsilon .$$

This parameter reflects the damping feature of NiTi SMAs, which is a unique property of the metals and has been extensively applied in engineering applications. 

To evaluate the proposed model, some experiments under the uniaxial cyclic loading conditions should be supplied in the following discussions and then used to verify the proposed model. Since the SMA bolt serving in the bolted joint in this research is always undertaking a tightened load, it implies that it is necessary to observe the cumulated deformation of the SMA under the cyclic loading by force form. Therefore, all cyclic tests in this study are controlled by axial load under cyclic tension-unloading and tension-tension with positive mean loads. The applied axial load corresponds to the axial nominal stress under the conditions of small strain prerequisite of the axial deformation of the specimen, e.g., less than 5%. The controlled axial load also corresponds to three kinds of the uniaxial nominal stress-controlled cyclic loading with positive mean axial stress, including cyclic tension-unloading tests with various applied peak stresses, e.g., 325 MPa and 365 MPa at room temperature, that are predicted by the proposed model. The number of cycles is prescribed as 50, and the stress rate is prescribed as 20 MPa/s. The superelastic NiTi SMA chosen for this study is comprised of 55.89 wt % nickel and 44.11 wt % titanium, from Xi’an Saite Metal Materials Development Co., Ltd., China, whose original phase is pure austenite at room temperature. The specimens employed in these tests are of diameter of 6 mm with gauge length of 30 mm machined from the as-received solid Nitinol bars. The material parameters used in the proposed model are determined by a trial-and-error method from the experimental data as described in Section 2.2, and are listed in Table 1 for the simulation. 

**Figure 3:**
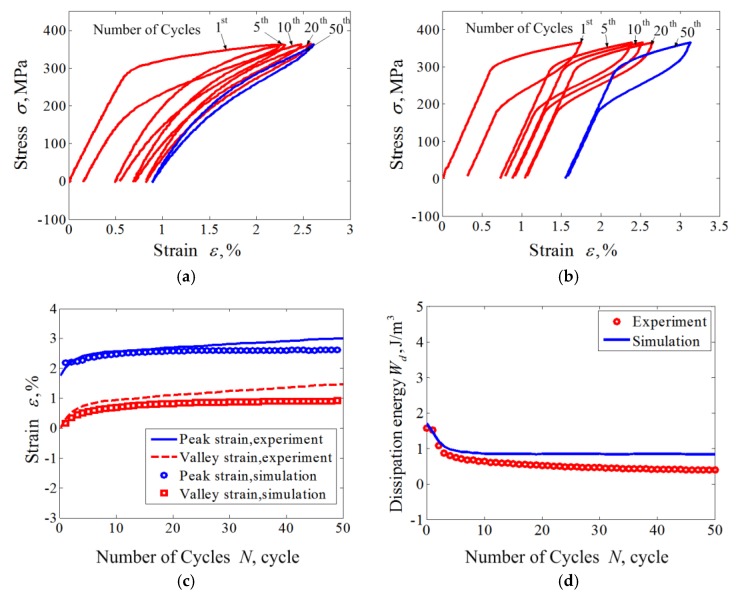
Experiments and simulations for cyclic tension-unloading transition ratchetting with constant loading stress of 365 MPa: (**a**) experimental results; (**b**) simulated results; (**c**) curves of peak and valley strains vs. cycle numbers; (**d**) curves of dissipation energy vs. cycle number.

The results obtained with various peak stresses are shown in Figure 2 and Figure 3. The figures show reasonable predictions by the proposed model as to the uniaxial transition ratcheting of the superelastic NiTi SMA, including the hysteresis loop curve, the evolutions of dissipation energy, and predicted peak and residual strain. The peak and valley strains (i.e., martensite residual strain) of the superelastic NiTi SMA increase progressively during the loading cycles to a stable value. The completely closed hysteresis loop means that the strain increment produced in the tension is completely recoverable during the subsequent unloading. The closed hysteresis loop still becomes smaller and smaller during the further cyclic loading, that means the dissipation energy decreases with the increasing number of cycle loadings.

Due to the introduction of the cosine nonlinear function in the phase transition model, the predicted results by the proposed model show an apparent nonlinear feature that is more coincidental with the experimental results compared with the numerical simulation results by [33]. Similarly, due to the introduction of a power function $c_{AM}^{f}(\sigma )$ that is associated with the applied loading level in simulating the phase transition ratcheting behavior, the model can reasonably predict the variation of peak strain and residual strain with different applied loading levels. 

From Figure 2c,d and Figure 3c,d, it can be seen that the ratcheting strains and dissipation energy *W_d_* decrease with an exponential function law during the cycle loadings. After certain numbers of cycles, both the ratcheting strains and dissipation energy show an apparent quick decrease to a stable value. These findings also agree with the conclusions presented by Kang [33]. 

**Figure 4:**
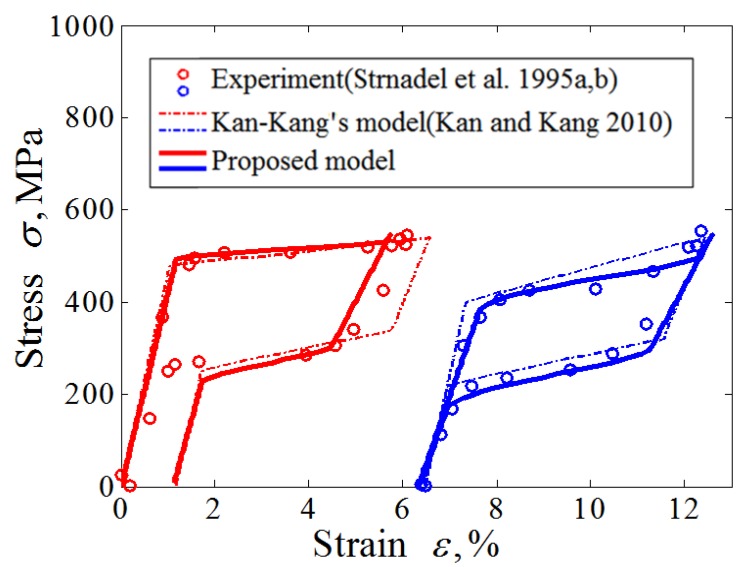
Stress-strain response of SMA to cyclic loading up to a constant value of stress: curves for the first and 50th cycles.

In order to further compare the proposed constitutive model with the existing cyclic constitutive model, Figure 4 gives the comparison of the predicted results of the proposed model and the existing model by Kan-Kang’s model [33]. Compared with the Kan-Kang’s model employing the linear phase transition hardening law, the proposed model can predict the nonlinear feature of the hysteresis loop with the same number of material parameters. Therefore, the proposed model has an obvious improvement compared with those models by Kan [33] in the prediction of the behavior of phase transition ratcheting and the nonlinear behavior of the hysteresis loop with consideration of the effect of the applied loading level. 

## 3. Finite Element Modeling for Self-Loosening of the SMA Bolted Joint

The preloading force and the irreversible strain accumulation of the NiTi SMA bolt have important influence on the self-loosening properties of bolted joints, which also play a crucial role in protecting the static and dynamic characteristics and the locking and sealing properties of the whole joint. For the normal material bolt, the self-loosening characteristic of the bolt is mainly determined by the magnitude of the clamping force of the bolt and the plastic accumulative properties of the material. However, for the NiTi SMA bolt, the self-loosening characteristics of the bolt is determined not only by the magnitude of bolt preload, but by the phase transition ratcheting effect of the NiTi SMA. The relative stiffness between the subparts and the overall stiffness of the bolted joint system has a strong influence on the preloading process and even the attenuation law of the bolt clamping force.

For the stiffness modeling and analysis of the NiTi SMA bolted joint system, not only the stiffness of the bolt bar, but the impact of the warping deformation of the bolt head and the contact stiffness of each contact surface of the bolted joint should be considered. However, since the warping deformation of the bolt head is relatively small compared with the large deformation of the SMA bolt bar, and the contact stiffness of the contact surface of the bolted joint is close to or even far beyond the stiffness of the bolt and members, the members combined with the contact surface show a deformation regularity of an approximately linear nature on the whole. Therefore, on the basis of the finite element model with the bar element of phase transition ratcheting of the NiTi SMA bolt in Section 2, the deformation coordination relationship should be established by just considering the deformation and stiffness properties of the NiTi SMA bolt bar and the members to study the influence law of member stiffness under the effects of the bolt preloading forces, sizes, and materials’ properties. Finally, the reasonability and effectiveness of the preload control strategy and the size design of the members should be demonstrated in specific load conditions.

### 3.1. Finite Element Modeling under Cyclic External Force Load

**Figure 5:**
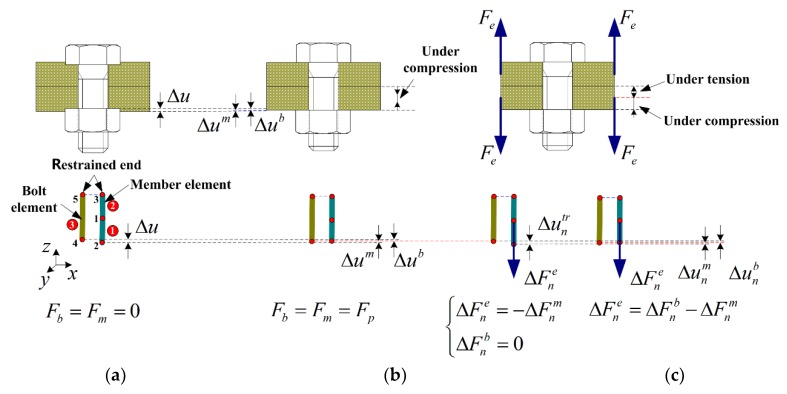
Loading process and finite element modeling of the bolted joint. (**a**) initial stage; (**b**) after preloaded; (**c**) cyclic loading stage.

During the self-loosening process of the NiTi memory alloy bolted joint under cyclic loading, the loading process to simulate the self-loosening of the bolted joint under cyclic loading should be divided into two stages, as shown in Figure 5. The first stage is the bolt preloading stage. In this research, a preset interference method that had successfully been used to simulate the preloading process of the bolt by [2,42,43] is adopted here to simulate the bolt preloading. The preset interference amount Δu is set up by shortening the distance of the bolt head and the nut in the model. That means that the interference will be produced between the bearing surfaces of the bolt head or nut and the member that will be integrated into one surface after bolt preloading.

The specific process is as follows: the initial parameters for simulation should be determined first, such as the elastic modulus of the material, cross sectional area, and effective length of the bolt bar, and the preloading force, to calculate the tensile stiffness of the bolt and the compression stiffness of the members. The finite element model with the bar element is established on the basis of the initial amount of preset interference $\Delta u_{0}$, which is the initial value of Δu. Since there is a symmetrical feature of the whole structure and load-bearing part of the bolted joint along the contact surface of the connected members, the finite element model is established by just a half of the whole bolted joint structure. The nodes (e.g., node numbers of 3 and 5) close to the bearing surface of one member are fixed as the restrained end.

During the iterative calculation process of applying the preloading force, the subtension force is applied on the bolt and the subcompression force is used on the member. The amount of interference Δu is reduced gradually. The amounts of deformation for each iterative substep are set as $\Delta u_{}^{b}$ and $\Delta u_{}^{m}$ for the bolt and member, respectively. The corresponding changes for the tension force of the bolt and the compression force of the member should also be calculated. For the case of Δu=0, which means the amount of penetration between the bearing surfaces of the bolt or nut and the member is zero, the iteration is terminated. At this time, the bearing surfaces of the bolt or nut and the member are attached to each other, and the tension force of the bolt is equal to the compression force of the member, i.e., $F_{b}=F_{m}=F_{p}$, which also means that the preloading process is finished. 

**Figure A1:**
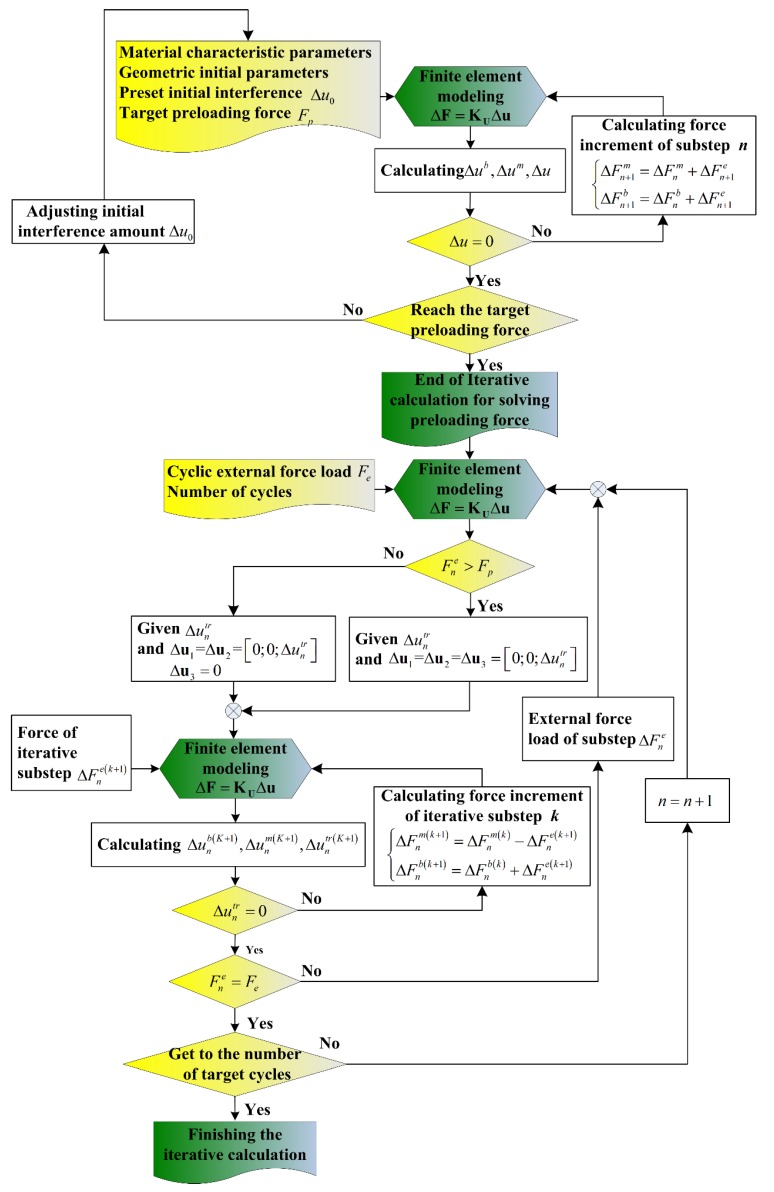
Flow chart of analysis to determine the preload and self-loosening of a bolt under cyclic external force load.

However, the preset amount of initial interference $\Delta u_{0}$ may not necessarily produce the aimed preloading force $F_{p0}$. The amount of initial interference $\Delta u_{0}$ should be continuously adjusted by a trial-and-error method to give the proper preloading force. The specific implementation process is shown in the flow chart of Figure A1. 

It should be noted that the NiTi SMA bolt element and the member element are independent of each other in finite element modeling. It means that the force increments of iteration for $\Delta F_{n}^{m}$ and $\Delta F_{n}^{e}$ are applied on the NiTi SMA bolt element and the member element, respectively, in spite of $\Delta F_{n}^{m}=\Delta F_{n}^{e}$. Then, the deformations for each element are calculated independently until the condition of convergence Δu=0 is satisfied. 

The equilibrium equation of the SMA bolt or member can be expressed using the standard finite element assembly operation:

(55) $$\Delta F=K_{U}\Delta u,$$

where ΔF indicates the increment of nodal force vectors of bar elements of the finite element model, Δu is the nodal displacement vectors, and $K_{U}$ is the stiffness matrix.

It should be noted that the members are in the compression status after preloading. Due to the effect of the external load $F_{e}$, the compression extent on the side of the bearing contact of the bolt head or nut increases, one on the side of the bearing contact of another member is released. Based on the discussions above, the finite element model of a member should be composed of two bar elements, as shown in Figure 5. It can be seen that the member elements consist of the nodes 1, 2, and 3. Two elements are connected by node 1. The node 3 is the restrained end, and the node 2 is the applied end of the force load of member element. Thus, Equation (55) can be further decomposed into

(56) $$\left[\begin{array}{c} \Delta F_{1}\\ \Delta F_{2}\\ \Delta F_{3} \end{array}\right]=\left[\begin{array}{ccc} K_{u}^{11} & K_{u}^{12} & K_{u}^{13}\\ K_{u}^{21} & K_{u}^{22} & K_{u}^{23}\\ K_{u}^{31} & K_{u}^{32} & K_{u}^{33} \end{array}\right][\begin{array}{c} \Delta u_{1}\\ \Delta u_{2}\\ \Delta u_{3} \end{array}],$$

where $u_{1}$, $u_{2}$, and $u_{3}$ and $F_{1}$, $F_{2}$, and $F_{3}$ are the displacements and the forces of nodes 1, 2, and 3, respectively. $F_{2}$ and $F_{3}$ are a pair of equilibrium forces with equal magnitude in opposite directions. $K_{u}^{11}$, $K_{u}^{12}$, $K_{u}^{13}$, $K_{u}^{21}$, $K_{u}^{22}$, $K_{u}^{23}$, $K_{u}^{31}$, $K_{u}^{32}$, and $K_{u}^{33}$ are the partitioned matrices of the matrix $K_{U}^{m}$.

Since the finite element model of a member is equivalent to a bar element, the relationship between the force vector and displacement vector of the node *i* that belongs to this bar element can be written as

(57) $$F_{i}=E_{k}A_{k}\varepsilon_{mk}\frac{x}{L_{k}}=\frac{E_{k}A_{k}}{L_{k0}}\left(L_{k}-L_{k0}\right)\frac{x}{L_{k}}=k_{mk}\left(L_{k}-L_{k0}\right)\frac{x}{L_{k}},$$

where $A_{k}$ is the equivalent cross-sectional area of the element *k*, and *k* = 1,2 for member elements. $L_{k0}$ is the initial unstressed length of element *k*, $E_{k}$ is the elastic modulus of element *k*, $\varepsilon_{mk}=\left(L_{k}-L_{k0}\right)/L_{k0}$ is the compression strain of member element *k*, and $k_{mk}$ is the compression stiffness of member element *k*. $F_{i}$ is the force vector of element *i* and $x=x_{i}-x_{j}$ depicts the relative nodal position denoted by $x_{i}$ and $x_{j}$ in the global frame. *i*, *j* = 1, 2, or 3 are the node numbers of member elements in equilibrium state. $L_{k}={\left[{\left(x_{i}-x_{j}\right)}^{T}\left(x_{i}-x_{j}\right)\right]}^{1/2}$ is the stressed length of the selected member element after deformation. $x/L_{k}$ is the nodal position vectors of member elements.

With the first-order Taylor expansion of Equation (57), it can be derived as

(58) $$\Delta F_{i}=\frac{\partial F_{i}}{\partial x}\;\Delta x.$$

Since $\Delta F_{j}=-\Delta F_{i}$, the incremental relationship between the nodal force and element length can be expressed as

(59) $$\Delta F_{k}=K_{mk}\Delta u_{k},$$

where $\Delta F_{k}=\left[\begin{array}{c} \Delta F_{i}\\ \Delta F_{j} \end{array}\right]$, $\Delta u_{k}=\left[\begin{array}{c} \Delta u_{i}\\ \Delta u_{j} \end{array}\right]=\left[\begin{array}{c} \Delta x_{i}\\ \Delta x_{j} \end{array}\right]$ is the incremental displacement vector of node *i* or *j*, and $K_{mk}=\left[\begin{array}{cc} k_{mk} & -k_{mk}\\ -k_{mk} & k_{mk} \end{array}\right]$. 

The stiffness matrix of one node can be obtained by the differential calculation as

(60) $$k_{mk}=\frac{\Delta F_{i}}{\Delta x}=\frac{E_{k}A_{k}}{L_{k}}\frac{xx^{T}}{L_{k}^{2}}+\frac{E_{k}A_{k}}{L_{k}}\frac{L_{k}^{}-L_{k0}^{}}{L_{k0}^{}}I_{3\times 3}=\frac{k_{mk}L_{k0}}{L_{k}}\frac{xx^{T}}{L_{k}^{2}}+k_{mk}\frac{L_{k}^{}-L_{k0}^{}}{L_{k}^{}}I_{3\times 3},$$

where $I_{3\times 3}$ is a 3 × 3 identity matrix.

The equilibrium equation of member structure can be achieved using the standard finite element assembly operation as follows:

(61) $$\Delta F=\sum_{k}K_{mk}\Delta u_{k}=K_{U}^{m}\Delta u,$$

where Σ is the standard finite element assembly operator and Δu is the incremental displacement set vector of $\Delta u_{k}$.

Noting the boundary conditions of the finite element model, $\Delta u_{3}=0$ and $\Delta F_{1}=0$ (since the force of node 1 is of the internal free force, no external load exits at this node) during the preloading of the bolt. Substituting these conditions into Equation (56) leads to the following relationship:

(62) $$\left\{ \begin{array}{l} K_{u}^{11}\Delta u_{1}+K_{u}^{12}\Delta u_{2}=0\\ K_{u}^{21}\Delta u_{1}+K_{u}^{22}\Delta u_{2}=\Delta F_{2}\\ K_{u}^{31}\Delta u_{1}+K_{u}^{32}\Delta u_{2}=\Delta F_{3} \end{array},\right.$$

Further solutions can be obtained as

(63) $$\left\{ \begin{array}{l} \Delta u_{1}={\left[K_{u}^{21.}-K_{u}^{22}{\left(K_{u}^{12}\right)}^{-1}K_{u}^{11}\right]}^{-1}\Delta F_{2}\\ \Delta u_{2}=-{\left(K_{u}^{12}\right)}^{-1}K_{u}^{11}\Delta u_{1} \end{array}.\right.$$

According to the above calculation, the deformation of each member element and the relationship of stress and strain are obtained.

As for the SMA bolt, the finite element model of the bolt is established simplistically to be a bar element. As shown in Figure 5, the SMA bolt element consists of nodes 4 and 5. Therefore, Equation (55) can be divided into

(64) $$\left[\begin{array}{c} \Delta F_{4}\\ \Delta F_{5} \end{array}\right]=\left[\begin{array}{cc} K_{u}^{44} & K_{u}^{45}\\ K_{u}^{54} & K_{u}^{55} \end{array}\right]\left[\begin{array}{c} \Delta u_{4}\\ \Delta u_{5} \end{array}\right],$$

where $u_{4}$, $u_{5}$ and $F_{4}$, $F_{5}$ are the displacements and the forces of nodes 4 and 5, respectively. $F_{4}$ and $F_{5}$ are a pair of equilibrium forces with equal magnitude in opposite directions. $K_{u}^{44}$, $K_{u}^{45}$, $K_{u}^{55}$, and $K_{u}^{54}$ are the partitioned matrices of matrix $K_{U}^{b}$. 

The boundary condition of the SMA bolt element during the preloading of the bolt is $\Delta u_{5}=0$. Thus,

(65) $$\left\{ \begin{array}{l} K_{u}^{44}\Delta u_{4}=\Delta F_{4}\\ \Delta F_{5}=-\Delta F_{4} \end{array}\right..$$

The further derivation gives

(66) $$\Delta u_{4}={\left[K_{u}^{44}\right]}^{-1}\Delta F_{4}.$$

Since the finite element model of the SMA bolt is established as just one bar element, there is no need to carry out the assembly calculation of finite element modeling. Therefore,

(67) $$\Delta F=K_{b}\Delta u=K_{U}^{b}\Delta u,$$

where $K_{b}=\left[\begin{array}{cc} k_{b} & -k_{b}\\ -k_{b} & k_{b} \end{array}\right]$ and $k_{b}=K_{U}^{sma}$. $K_{U}^{sma}$ is the stiffness matrix of a node in the SMA bolt element, and can be seen in Equation (52). It can be given by comparing Equation (64) and Equation (67):

(68) $$\Delta F=\left[\begin{array}{c} \Delta F_{4}\\ \Delta F_{5} \end{array}\right],\;\Delta u=\left[\begin{array}{c} \Delta u_{4}\\ \Delta u_{5} \end{array}\right],K_{U}^{b}=[\begin{array}{cc} K_{u}^{44} & K_{u}^{45}\\ K_{u}^{54} & K_{u}^{55} \end{array}].$$

The second stage of the loading process, as shown in Figure 5, is to simulate the self-loosening of the bolted joint under the cyclic external load $F_{e}$. According to the previous description, the finite element model of one member consists of two bar elements. The external load $F_{e}$ should be applied to the node 1 that connects two bar elements. 

The members are in the compression status after being preloaded. Due to the effect of the external load $F_{e}$, the compression degree on the side of the bearing contact of the bolt head or nut increases and one on the bearing contact side of another member is released. Based on the discussion above, the finite element model of a member should be composed of two bar elements. Under the effect of this load, the element 4 is tensioned and the element 2 is undertaking compression. This modeling method is to reasonably simulate the practical status of the bolted joint. 

The specific iteration process is described as follows. Under the effect of external load, the calculation process is divided into two steps for a single iterative substep. The external increment force $\Delta F_{n}^{e}$ at substep *n* is firstly applied to the concatenation node of two member bar elements. The nodes 3 and 5 are fixed as restriction ends. At this time, the bar element 2 is tensioned in this substep (i.e., $\Delta F_{n}^{m}=\Delta F_{n}^{e}$). The distance between nodes of element 1 is kept constant. Hence, $\Delta F_{n}^{b}=0$. It means that the preloading of bolt element 1 remains constant. Under the effect of the external increment force $\Delta F_{n}^{e}$ at substep *n*, the displacement $\Delta u_{n}^{tr}$ between the nodes 2 and 4 of elements 1 and 3 at the side of the bearing contact surface of the bolt head or nut is produced. 

Since the surfaces between the bolt head or nut and the member should be practically in contact and attached to each other, this means that the nodes 2 and 4 should be entirely coincident in the absolute coordinate system. Therefore, under the effect of the external increment force, the second step during the iterative calculation of a single substep is to ensure that they are coincident. The specific implementation process is similar to the calculation process of bolt preloading. As per the previous description, after the external increment force $\Delta F_{n}^{e}$ is applied, the amount of interference $\Delta u_{n}^{tr}$ is produced between the nodes 2 and 4. Then, an embedded iteration calculation should be carried out in the following step. A tension force $\Delta F_{n}^{e\left(k+1\right)}$ is applied to the SMA bolt element, and the same magnitude of compression force $\Delta F_{n}^{e\left(k+1\right)}$ with the opposite direction of tension force $\Delta F_{n}^{e\left(k+1\right)}$ is applied to the member element. It can be expressed as

(69) $$\left\{ \begin{array}{l} \Delta F_{n}^{m\left(k+1\;\right)}=\Delta F_{n}^{m\left(k\right)}-\Delta F_{n}^{e\left(k+1\right)}\\ \Delta F_{n}^{b\left(k+1\right)}=\Delta F_{n}^{b\left(k\right)}+\Delta F_{n}^{e\left(k+1\right)} \end{array}\right.$$

During that iterative calculation, the amount of interference $\Delta u_{n}^{tr}$ gradually reduces. The updated $\Delta u_{n}^{b}$ and $\Delta u_{n}^{m}$ are also obtained. As $\Delta u_{n}^{tr}=0$, the embedded iteration is in termination. At this time, the bearing surface of the bolt head or nut and that of a member are integrated into one surface. The bolted joint system reaches a new equilibrium state, and satisfies the following equation:

(70) $$\Delta F_{n}^{e}=\Delta F_{n}^{b}-\Delta F_{n}^{m},$$

According to the descriptions above, under the effect of the external load $F_{e}$, there exist two contact status between the contact surface of members.

① As the external load $F_{e}$ is smaller than the initial preloading force of bolt $F_{p0}$, the system will satisfy the force equilibrium relationship as $F_{b}=F_{e}+F_{m}$.
② As the external load $F_{e}$ is larger than the initial preloading force of bolt $F_{p0}$, $F_{m}=0$ is satisfied at this time. Then, the system will satisfy the force equilibrium relationship as $F_{b}=F_{e}$. Meanwhile, the constraint at the node 3 is removed and the element 2 is in the state of no stress.

It can be seen from the discussions about the boundary conditions of finite element modeling that the internal force of node 1 satisfies $\Delta F_{1}=0$ when the external load is absent, and that satisfies $\Delta F_{1}=\left[0;0;\Delta F_{n}^{e}\right]$ when the external load exists. 

As previously described, the second stage of the loading process of the finite element model that simulates the self-loosening of a bolted joint under a cyclic external load $F_{e}$ should be divided into two steps. For the first step, the external increment force $\Delta F_{n}^{e}$ at step *n* is applied to the concatenation node of two member elements, and $\Delta u_{1}=\Delta u_{2}$. At the same time, the node 3 is always in the state of restraint as $\Delta u_{3}=0$. Therefore, the following relationship can be derived as

(71) $$K_{u}^{11}\Delta u_{1}+K_{u}^{12}\Delta u_{2}=\Delta F_{1}$$

The following equation can be given with further derivation as

(72) $$\Delta u_{1}=\Delta u_{2}={\left(K_{u}^{11\;}+K_{u}^{12}\right)}^{-1}\Delta F_{1}$$

After the first step above is finished, there is an amount of interference $\Delta u_{n}^{tr}$ existing between the nodes 2 and 4. However, the absolute coordinates of these two nodes should always be consistent. Therefore, the second step with embedded iterative calculation should be carried out, the process of which is similar to the first stage of the loading process of the finite element model that means the bolt preloading stage. 

With regard to the first step of the second stage, as the condition $F_{e}>F_{p}$ is satisfied, the nodes of bar element will move simultaneously within the limited substep time. At present, there is no increment of internal force for the nodes of those elements. Therefore, it is assumed that the displacement increment satisfies the following equation as $\Delta u_{1}=\Delta u_{2}=\Delta u_{3}=\left[0;0;\Delta u_{n}^{tr}\right]$. For the second step of this stage, the embedded iterative calculation with a similar process to the bolt preloading stage should be carried out to solve the equilibrium force of subparts of the bolted joint system. The convergence conditions for the iteration of this step is $F_{n}^{e}=F_{n-1}^{e}+\Delta F_{n}^{e}=F_{e}$, and $F_{b}=F_{e}$ at that moment. For the case of unloading of zero external force, the convergence condition is that the internal forces of two member elements are equal.

### 3.2. Finite Element Modeling under Cyclic External Displacement Load

**Figure A2:**
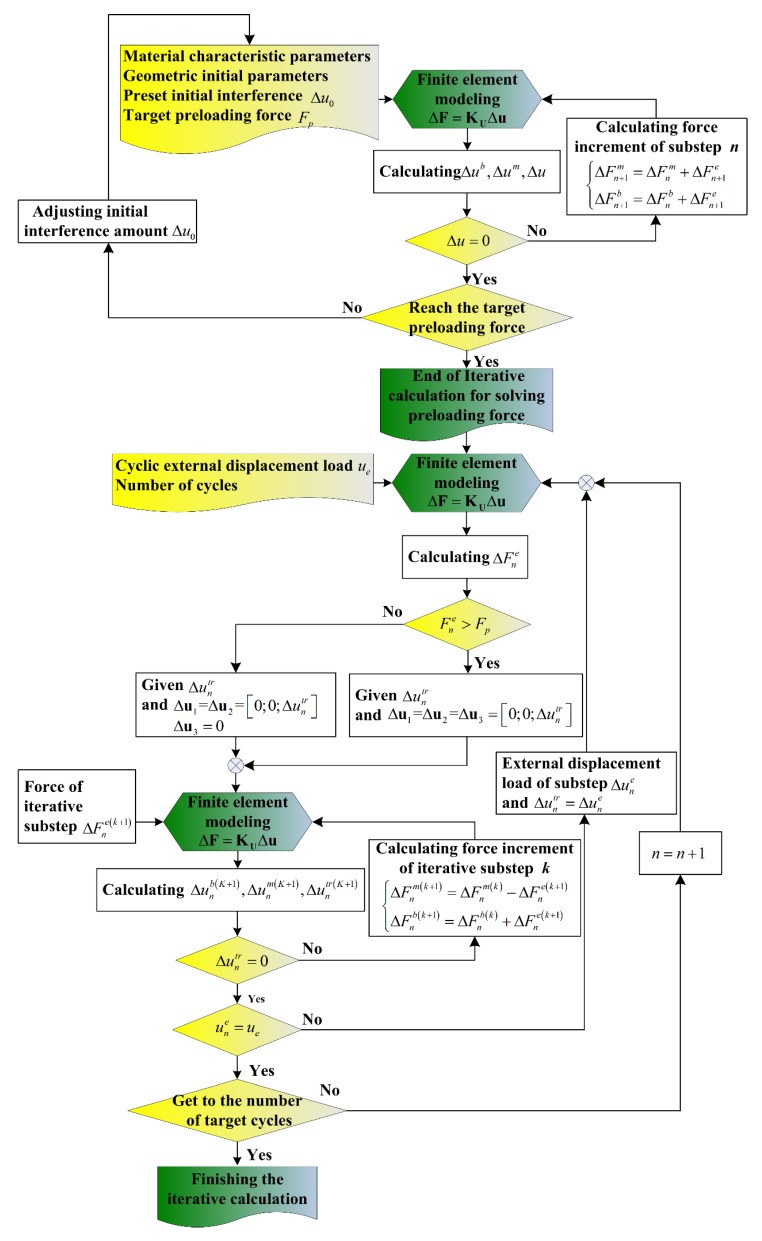
Flow chart of analysis to determine the preload and self-loosening of a bolt under cyclic external displacement load.

The finite element modeling of the self-loosening process of a bolt is closely related to the external load form. The finite element modeling method of the self-loosening of a bolt under a cyclic external force load has been described before. That modeling method under a cyclic external displacement load should also be discussed in this work. The calculation process can also be divided into two stages. The specific implementation process can be seen in the flow chart of Figure A2. In the first preloading stage, the modeling method is identical with that under a cyclic external force load. The main difference in modeling method for the different external loading form is in the second stage. As the external load is of the force form, the deformation variable $\Delta u_{n}^{tr}$ can be resolved by the finite element method in that premise of applying the external force load $\Delta F_{n}^{e}$ at substep *n*. However, as the external load is of the displacement form, the variable needed to be resolved is the substep of external force $\Delta F_{n}^{e}$ with the given incremental displacement of $\Delta u_{n}^{tr}$ at substep *n*. The stopping conditions for convergence of the current iteration of this step is $u_{n}^{e}=u_{n-1}^{e}+\Delta u_{n}^{e}=u_{e}$.

## 4. Results and Discussion

The self-loosening of the SMA bolt will be discussed in this section on the basis of the finite element model of the NiTi SMA bolted joint established in the last section. The M6 NiTi SMA bolt is firstly selected for this research. Upper and lower members are completely identical, including dimensional size and materials. Motosh and Nassar [44,45] had developed the analytical model to describe the member stiffness precisely, which is adopted here to calculate the stiffness value as 4 × 10^8^ N·m^−1^ with the dimensional sizes of height 50 mm and diameter 30 mm, and the elastic modulus 70 × 10^9^ Pa and Poisson ratio 0.28 of selected aluminum.

**Figure 6:**
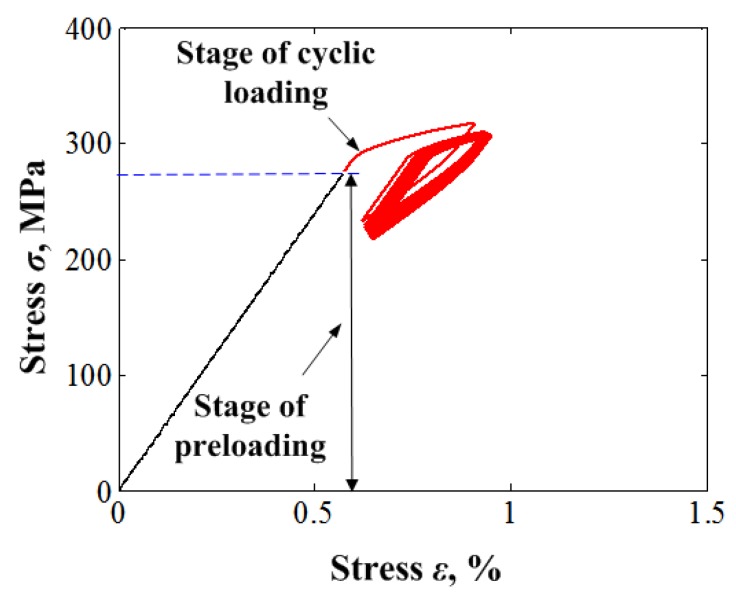
Curves of stress-strain responses on bolt bar.

Finite element simulations are conducted for several loading cases. For each loading case, a total of up to 20 transverse loading cycles is simulated after the initial preloading of the bolt is applied. Figure 6 shows the stress-strain hysteresis loops obtained from the bolt bar under cyclic displacement loading with the final condition of zero external force. Obviously, the SMA material of the bolt experienced the cyclic ratcheting with increasing number of loading cycles. The stress in this position keeps decreasing, while the strain at the same one increases with increasing number of transverse loading cycles due to cyclic strain ratcheting. The increasing rate of accumulation of peak strain is lower than that of valley strain, and the decreasing rate of accumulation of peak stress is simultaneously lower than that of valley stress. 

**Figure 7:**
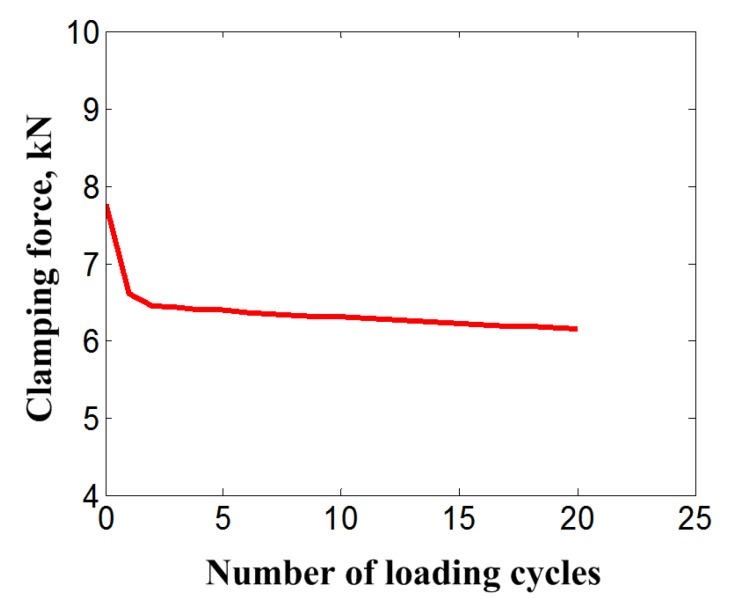
Clamping force reduction of bolt with increasing loading cycles.

The clamping force can be calculated through the integration of the axial stresses across the cross section of the bolt. The variation of clamping force with the loading cycles can be obtained. Figure 7 shows the clamping force reduction with increasing loading cycles obtained from the simulation results. It can be found that the attenuation rate of the clamping force of the SMA bolt gradually decreases and tends to be stable within a very small value after five loading cycles. The reduction of clamping force mainly happens in the initial several loading cycles. It decreases approximately 17.9% during the initial two cycles, which accounts for nearly 84% of the total loosening amount after the completion of loading cycles. This force reduction is mainly caused by the accumulation of martensite residual strain or the phase transition ratcheting. This mechanism causes the amount of effective elastic strain of the bolt bar to be gradually reduced at the end time point of one cycle loading, and leads to the clamping force reduction. Although the change of elastic modulus, which is affected by the martensite volume fraction and changed with the accumulation of residual martensite, has an influence on the clamping force reduction, this influence factor can be nearly ignored by comparison with that of residual martensite accumulation.

**Figure 8:**
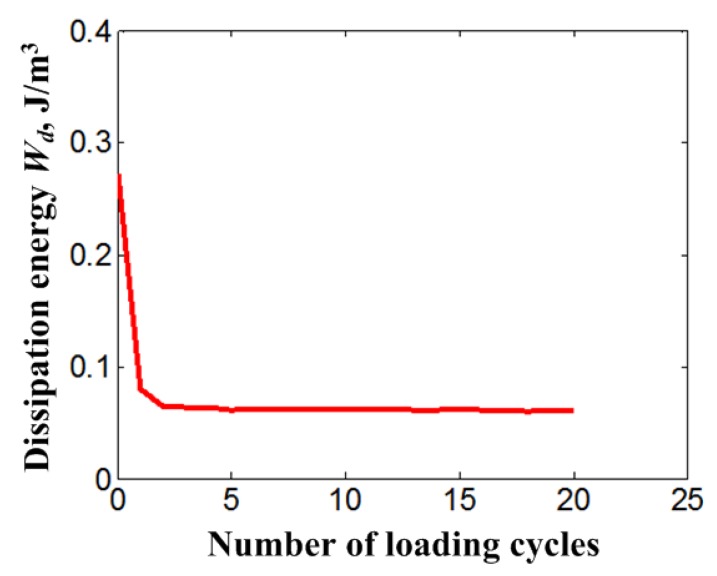
Dissipation energy (*W_d_*) reduction of bolt with increasing loading cycles.

It can be found from Figure 8 that the dissipation energy *W_d_* calculated across the area around the stress-strain curve of the SMA bolt bar in loading-unloading cycles decreases with an exponential function law during the stress cycle loading. Similar to the decrease of dissipation energy of a uniaxial cycle loading test, that of an SMA bolt also shows an obvious quick decrease to a stable value after a certain number of cycles. The remarkable decrease of dissipation energy means that the damping capability of superelastic NiTi SMA, which was also discussed in [46], degrades with the residual martensite accumulation and finally causes the functional failure of the NiTi SMA bolt. 

**Figure 9:**
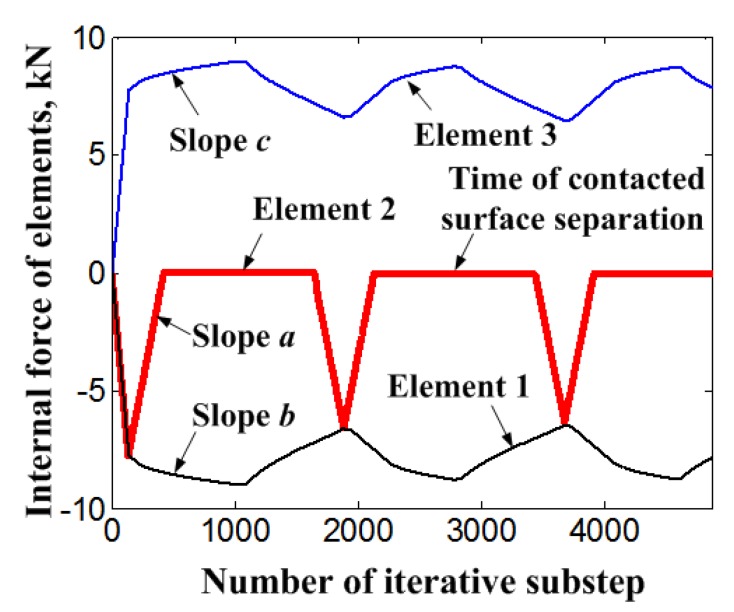
Internal force of elements with the increasing number of loading steps.

Figure 9 shows the change of internal force of each element with the increasing number of loading steps. It can be seen that the internal force of the bolt element 3 fluctuates with the loading cycles, and it is a counterforce against the member element 1. At the same time, after the beginning of the external displacement load for each cycle, the NiTi SMA bolt goes into the phase transition stage rapidly, with low stiffness and great deformation under even a small external force. That leads to a rapid attenuation of the internal force of the element 2 and the separation of the contact surface of members. After separation, the internal force of element 2 becomes zero. Based on the comparison of slopes *a*, *b*, and *c* in Figure 9, the slopes *b* and *c* reflect the change of internal force of the NiTi SMA bolt with increasing number of loading steps. The rate of change of slopes *b* and *c* is obviously smaller than that of slope *a*, and has nothing to do with the evolution of slope *a*. During the unloading process of the cyclic displacement load, the internal force of member element 2 increases rapidly. When the internal forces of the member elements 1 and 2 are equal, the embedded iteration of the single load step is finished.

**Figure 10:**
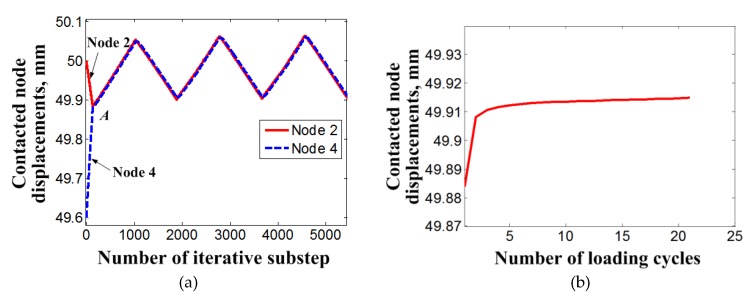
Variation of displacement of nodes connecting the bolt and member during the loading cycles: (**a**) change with the iterative substeps; (**b**) change with the loading cycles.

Figure 10a presents the variation of displacement of nodes 2 and 4 during the three loading cycles. Since the preset interference method is used to simulate the preloading of the bolt, there exists a preset distance between the nodes 2 and 4 in the initial stage of the simulation. After finishing the bolt preloading, the nodes 2 and 4 meet at point A. Due to the stiffness of the member selected in this research being higher than that of the SMA bolt, the slope of displacement change of node 2 is lower than that of node 4. These two slopes maintain identical change in the following loading cycles. Figure 10b shows a clearer description of the displacement of the connected node at the finished point of each loading cycle step. It can be seen that the node displacement gradually increases with the loading cycles due to the accumulation of the residual martensite of the SMA bolt. Similar to the change laws of the clamping force of the bolt and its dissipation energy, the increasing rate of the displacement of the connected node mainly takes place in the initial several loading cycles and tends to be a small, stably increasing value after that. The amount of node displacement for the initial two cycles is 0.004 mm, which accounts for approximately 89% of the total loosening amount after the completion of loading cycles. The stability slope of the node displacement increment is about 0.02%.

**Figure 11:**
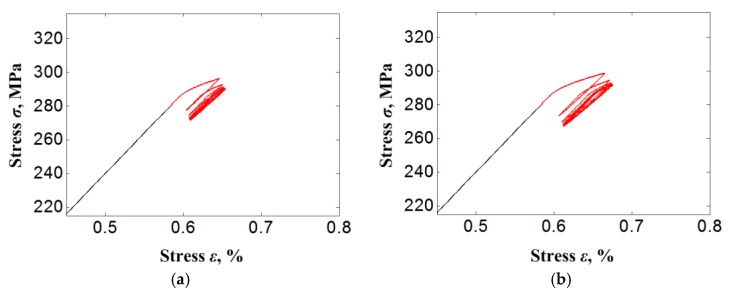

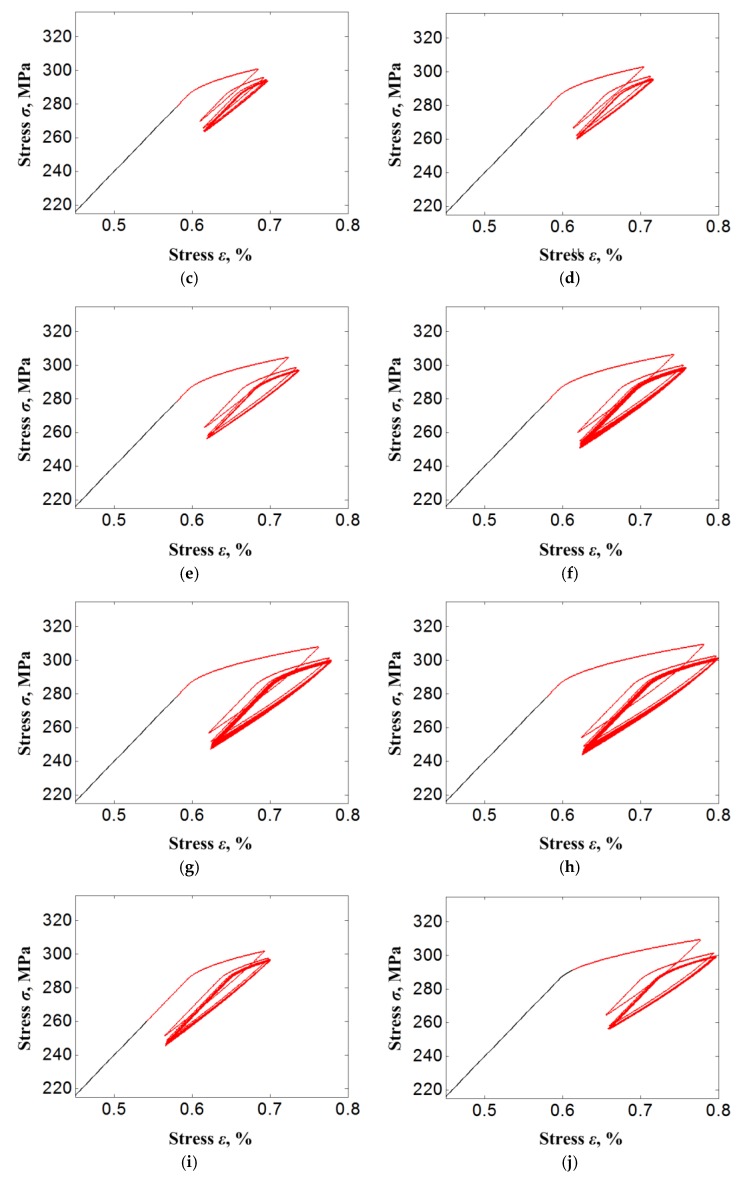
Curves of stress-strain responses on bolt bar under different load cases. (**a**) *P*_0_ = 7.9 kN, *u_e_* = 0.036 mm; (**b**) *P*_0_ = 7.9 kN, *u_e_* = 0.046 mm; (**c**) *P*_0_ = 7.9 kN, *u_e_* = 0.056 mm; (**d**) *P*_0_ = 7.9 kN, *u_e_* = 0.066 mm; (**e**) *P*_0_ = 7.9 kN, *u_e_* = 0.076 mm; (**f**) *P*_0_ = 7.9 kN, *u_e_* = 0.086 mm; (**g**) *P*_0_ = 7.9 kN, *u_e_* = 0.096 mm; (**h**) *P*_0_ = 7.9 kN, *u_e_* = 0.106 mm; (**i**) *P*_0_ = 7.4 kN, *u_e_* = 0.086 mm; (**j**) *P*_0_ = 8.3 kN, *u_e_* = 0.086 mm.

Figure 11 shows the curves of stress-strain responses on the bolt bar under different displacement load magnitudes with identical preloading of the bolt of 7.9 kN, and the different preloading of the bolt with the identical displacement load of 0.086 mm. It is clear that all stress-strain responses show an obvious superelastic hysteresis loop characteristic. This characteristic is exhibited more obviously with the magnitude increase of displacement load or preloading of bolt.

**Figure 12:**
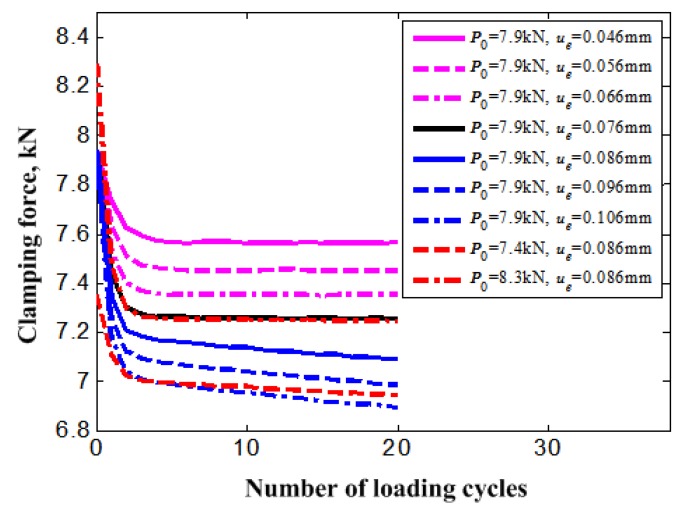
Clamping force reduction of bolt with increasing loading cycles under different load cases.

From Figure 12, the clamping force of the bolt shows obvious reduction during the initial five cycle loadings for all of the loading cases and gradually attenuates to a stable increment. In general, the attenuation rate of the bolt clamping force increases with the magnitude increase of displacement load and preload. The loading case for *P*_0_ = 7.9 kN and *u_e_* = 0.076 mm seems to be the demarcation line for the attenuation rate of the bolt clamping force. For the loading case of larger displacement loads than *u_e_* = 0.076 mm, the attenuation rate of the bolt clamping force shows an obvious increase than those of lower displacement loads. The change for the attenuation rate of the bolt clamping force is caused by the division of the contact surface of members under the loading cases of larger displacement loads than *u_e_* = 0.076 mm. This reason can also be used to explain why the larger preload case for *P*_0_ = 8.3 kN produces a lower attenuation rate of bolt clamping force, to the contrary.

**Figure 13:**
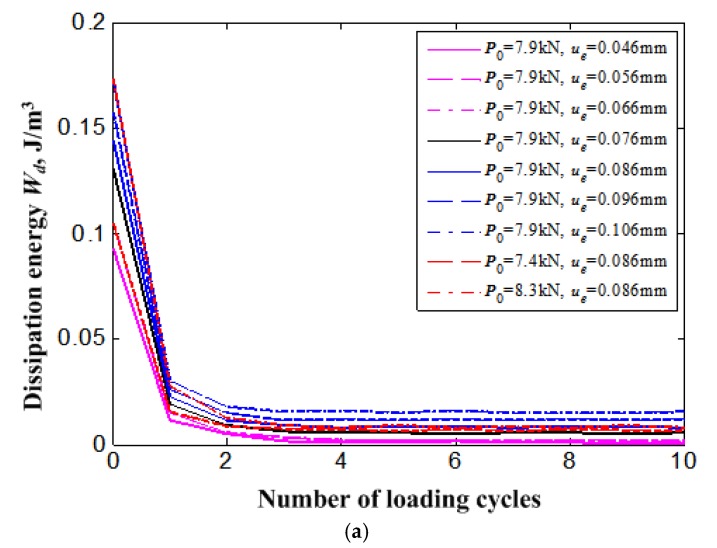

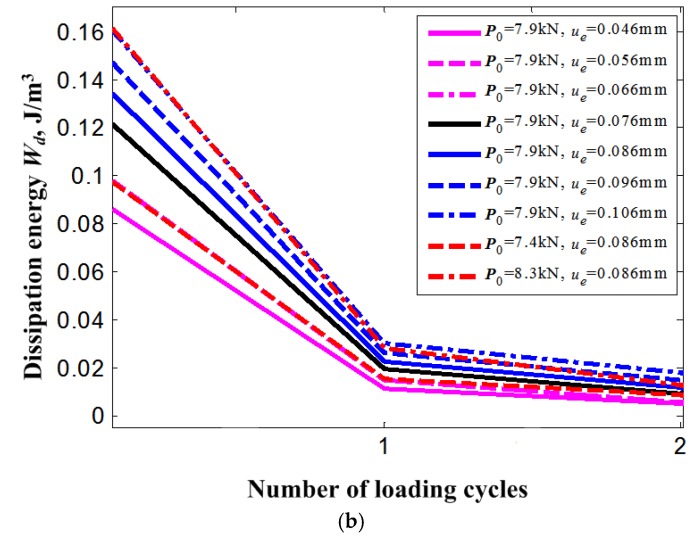
Dissipation energy (*W_d_*) reduction of bolt for all loading cases with increase of loading cycles: (**a**) for all cycles; (**b**) for the initial two cycles.

Figure 13 is the dissipation energy *W_d_* of the SMA bolt bar during the loading cycles. The dissipation energy of the SMA bolt also shows an obvious quick decrease to a stable value after a certain number of cycles. Obviously, the dissipation energy of the SMA bolt will increase with the increase of external displacement load that corresponds to the area around the stress-strain curve of the SMA bolt bar in loading-unloading cycles as shown in Figure 11. It can also be found that the dissipation energy of the SMA bolt increases with the increase of bolt preload. It may provide a beneficial reference for the engineer that a relatively higher preload force of the SMA bolt will increase the antivibration capability of the damping structure with the SMA bolted joint.

## 5. Conclusions

In this work, a macroscopic constitutive model of superelastic shape memory alloys is established to simulate the uniaxial transition ratcheting behaviors of the superelastic SMA undergoing cyclic loading. A finite element model is then derived to analyze the self-loosening behavior of the superelastic SMA bolt based on the proposed constitutive model. The curves of stress-strain responses on the bolt bar, clamping force reduction law, and dissipation energy change law of the bolted joint are discussed for different external loading cases and preload forces of the bolt. It is found that the great attenuation of the clamping force and dissipation energy of the SMA bolt happens during the initial several loading cycles and tends to be stable with a very small value afterward. The attenuation rate of the bolt clamping force shows obvious increase caused by the division of the contact surface of members for a larger displacement load than a certain value (e.g., *u_e_* = 0.076 mm). The conclusion that the dissipation energy of the SMA bolt increases with a relatively higher preload force of the SMA bolt, which will be beneficial to increase the antivibration capability of the damping structure with the SMA bolted joint, could provide a beneficial reference for engineering design.

## Figures and Tables

**Table 1:** Material parameters determined by tests of the current work.

$E_{A}^{}$ = 48 GPa	$E_{M}^{}$ = 35 GPa	$\nu_{A}^{}$ = 0.3	$\nu_{M}^{}$ = 0.3	$\varepsilon_{L}^{}$= 0.063	T= 295 K
$\sigma_{s0,T}^{AM}$= 285 MPa	$\sigma_{f0,T}^{AM}$= 458 MPa	$\sigma_{s0,T}^{MA}$= 345 MPa	$\sigma_{f0,T}^{MA}$= 164 MPa		
$\sigma_{s1,T}^{AM}$= 225 MPa	$\sigma_{f1,T}^{AM}$= 458 MPa	$\sigma_{s1,T}^{MA}$= 310 MPa	$\sigma_{f1,T}^{MA}$= 125 MPa;		
$c_{s}^{AM}$= 0.05	$c_{f}^{AM}$= 0.05	$c_{s}^{MA}$= 0.05	n = 3	$\xi_{\max}^{ir}$ = 0.84	b = 0.5
